# Crystallized Domain Walls in HfO_2_ Enable Chessboard‐Type Ultra‐High‐Dense Ferroelectric Memory

**DOI:** 10.1002/advs.77599

**Published:** 2026-09-13

**Authors:** Pawan Kumar, Hyun‐Jae Lee, Yungyeom Kim, Jihoon Choi, Jun Hee Lee

**Affiliations:** ^1^ Department of Energy Engineering School of Energy and Chemical Engineering Ulsan National Institute of Science and Technology (UNIST) Ulsan Republic of Korea; ^2^ Graduate School of Semiconductor Materials and Devices Engineering Ulsan National Institute of Science and Technology (UNIST) Ulsan Republic of Korea

**Keywords:** condensed matter, domain wall crystals, ferroelectrics, semiconductors, ultra‐high‐dense memory

## Abstract

Ferroelectric domain walls (DWs) are interfacial buffering regions between oppositely polarized domains, whose stability and density critically influence the endurance and scalability of ferroelectric memories. In conventional ferroelectrics, accommodating oppositely polarized regions requires DWs to spread over several unit cells through a high‐symmetry transition region, incurring substantial energetic costs and limiting device scalability. Here, the reported DWs in HfO_2_ are sharply localized within nearly a single‐unit‐cell width as low‐symmetry structures, resulting in remarkably low DW energies along arbitrary crystallographic directions. Strikingly, these DWs exhibit distinct low‐energy crystalline configurations, including the experimentally observed *Pbca* DW structures and previously unreported phases in HfO_2_. The sharply localized DWs ultimately stabilize ultrasmall square sub‐nm^2^ ferroelectric domains separated by sizable energy barriers, thereby enabling chessboard‐type ultra‐high‐density domains reaching ∼400 *Tbit*/*cm* ^2^. Inspired by experimentally observed anomalous behaviors such as sluggish DW dynamics and imprint effects, our discovery establishes that DWs formed during polarization switching can themselves constitute distinct and stable bulk‐like structural phases, expanding the conventional paradigm of DWs into a new structural building block for emergent phases. Consequently, this discovery provides a streamlined route toward enhanced ferroelectric functionality and improved CMOS‐compatible device performance, including lower operating voltages and ultra‐high‐density memories.

## Graphical abstract

In general, DWs in ferroelectrics stabilize within a widely spread transition region between two oppositely polarized domains, which severely limits the achievable memory density. Here, the reported DWs in HfO_2_ constitute perfect crystalline structures along arbitrary crystallographic directions, enabling the formation of the narrowest square‐shaped chessboard‐type domains. This unique characteristic could enable ultra‐high‐density memory with capacities up to ∼400 *Tbit*/*cm* ^2^.

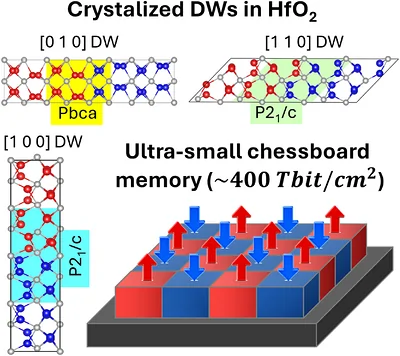

## 1 Introduction

In ferroelectric materials, domain walls (DWs) [1, 2] are nanoscale interfaces that separate regions with different orientations of spontaneous polarization and play a critical role in governing functional behavior rather than acting as passive boundaries. They strongly influence key physical properties [3, 4] such as dielectric permittivity [5], piezoelectric response [6, 7], and polarization switching behavior [8, 9], and are highly mobile, allowing their positions to be manipulated by external electric fields and other perturbations [10, 11]. The formation, stability, and mobility of DWs in ferroelectrics provide key technological advantages, making them highly promising for applications in non‐volatile memories [12, 13], next‐generation nanoelectronics [14], artificial intelligence [15], and neuromorphic computing [16]. In general, ferroelectric DWs are observed as spatially extended transition regions that locally adopt higher‐symmetry structures and reside in higher‐energy states compared to the surrounding domains [17]. This intrinsic energetic cost and spatial spreading can significantly limit achievable memory density [18, 19] and compromise the long‐term stability of domain configurations, posing fundamental challenges for ferroelectric‐based memory applications.

The discovery of ferroelectricity in HfO_2_ [20] has attracted significant interest due to its robust stability down to sub‐nanometer thin films [21], scale‐free ferroelectric behavior [22], and compatibility with complementary metal–oxide–semiconductor (CMOS) technology [23]. Importantly, fatigue‐free behavior [24], enhanced ferroelectricity [25], and lower coercive fields [26, 27] have been reported in HfO_2_‐based ferroelectrics, which further enhance their potential for technological applications. Notably, DWs in HfO_2_ can be stabilized within nearly half a unit‐cell width [22], offering a pathway toward high memory density. These DWs exhibit unconventional characteristics that do not exist in the bulk ferroelectric state and have recently been shown to retain a well‐defined crystalline antipolar *Pbca* structures even at the atomic scale [28, 29, 30]. Previous studies have reported unusual DW dynamics [31, 32], often described as sluggish DWs, referring to the tendency of once‐formed DWs to propagate only with difficulty [22]. This distinctive behavior highlights the importance of identifying the microscopic origins of DW stability and mobility in this material. Despite these advances, an atomic‐scale understanding of DWs along arbitrary crystallographic directions and their energetics in HfO_2_ remains lacking, although it is essential for achieving reliable ferroelectric scaling and enabling domain‐wall‐driven design of novel crystal structures beyond the bulk.

In this manuscript, we report 180° DWs in HfO_2_ that exhibit low‐symmetry, low‐energy structures along all crystallographic directions. DWs along the [0 1 0] direction adopt antipolar *Pbca* structures, whereas those along the [1 0 0] and [1 1 0] directions exhibit the antipolar monoclinic *P*2_1_/*c* phases. Notably, the *P*2_1_/*c* phases associated with the [1 0 0] and [1 1 0] directions are structurally distinct, although they share the same metastable energy, and both differ from the ground‐state *P*2_1_/*c* phase. These two distinct metastable *P*2_1_/*c* phases play a crucial role in stabilizing the narrowest possible DWs along arbitrary crystallographic directions and, to the best of our knowledge, one of them (emerging at [1 1 0] direction DWs) is reported here for the first time. We further demonstrate that the domain wall energies (DWEs) and structures for the [1 0 0], [0 1 0], and [1 1 0] directions can serve as reliable descriptors to predict DWs along any direction. Our phonon modes and their band curvature analyses reveal the origin of these DW structures and their associated energies. Furthermore, we show that these low‐symmetry and low‐energy DW configurations enable the stabilization of DWs confined in sub‐nm^2^ area in HfO_2_, enabling the chessboard‐type ultrahigh memory density of ∼400 $Tbit/cm^{2}$. Surprisingly, each domain is separated by sizable energy barriers across the crystallographic directions that prevent collapse and ensure long‐term stability. Given the growing importance of ferroelectric HfO_2_ devices, we expect that our findings will contribute to improving device performance by advancing the understanding of DW dynamics.

## 2 Results and Discussion

### 2.1 Domain Wall Topology in Ferroelectric HfO_2_

The widely recognized ferroelectric orthorhombic *Pca*2_1_ phase of HfO_2_ can switch its polarization (=  41 µ*C*/*cm* ^2^, estimated by using Equation S1) from the up‐polarized state (*U* phase) through four distinct pathways, leading to four symmetrically equivalent down‐polarized phases, denoted *D_xy_*, *D_y_*, *D_x_*, and *D* (Figure S1a), the subscript x and y in down‐polarized phases represent the reversal of unstable $X_{2}^{\prime }$ and antipolar $Y_{5}^{z}$ modes, respectively. Thus, four types of 180° DWs with distinct topology, namely *U*/*D_xy_*, *U*/*D_y_*, *U*/*D_x_*, and *U*/*D*, (Figure S1b–e) can form along any crystallographic direction. Among them, the *U*/*D_y_*, *U*/*D_x_*, and *U*/*D* DWs exhibit conventional behavior [33], as changes in spacer and ferroelectric layer arrangements, along with oxygen and Hf displacements during domain wall formation, drive them toward high‐symmetry, high‐energy states.

**FIGURE 1:**
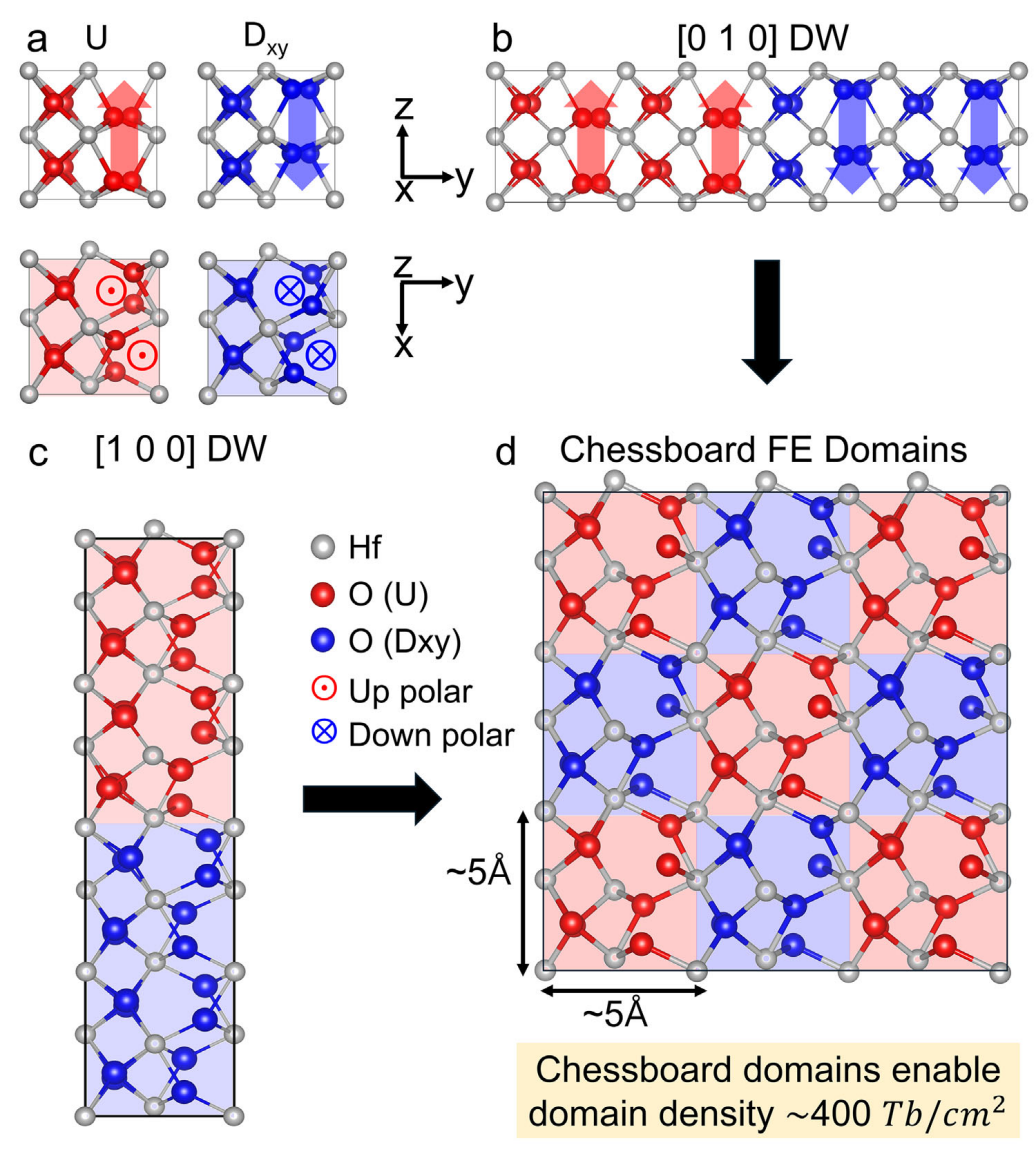
Topology of distinct DWs and possible ultrasmall square domains in HfO_2_. (a) Atomic structures of the *U* and *D_xy_* phases of *Pca*2_1_ HfO_2_ shown from different crystallographic views. Atomic structures of the DWs along the [0 1 0] direction (b) and the [1 0 0] direction (c), together with chessboard‐type domains at the individual unit‐cell scale (d), which enable an ultrahigh domain density of ∼400 *Tbit*/*cm* ^2^. Red and blue arrows indicate up and down polarization, respectively, while red‐ and blue‐shaded unit cells represent the *U* and *D_xy_* unit cells, respectively.

In contrast, we found that *U*/*D_xy_* DWs (Figure 1), in which polarization switching occurs solely through oxygen displacements within the ferroelectric layer without perturbing the surrounding domain‐wall environment (Figure S2), exhibit remarkable topological properties along the [0 1 0] (Figure 1b) and [1 0 0] (Figure 1c) directions, which give rise to the chessboard‐type ultranarrow square domains at the individual unit‐cell scale (Figure 1d). Thus, each unit cell can potentially store a memory bit, having in‐plane lattice constants of ∼5 Å, resulting in an exceptionally high memory density (=  *bit*/*Area*) of ∼400 *Tbit*/*cm* ^2^. In the following manuscript, we systematically investigate the energetics, structures, and ferroelectric properties of DWs along each direction, elucidate their microscopic origins, and their generalization in arbitrary in‐plane crystallographic direction.

### 2.2 Domain Walls Along [0 1 0] Crystallographic Direction

First, we analyze the experimentally observed [28, 29], well‐known [0 1 0] DWs in HfO_2_, where the lateral arrays of ferroelectric layers are oppositely oriented across the wall. Our atomic structural analysis shows that, in *U*/*D_xy_* [0 1 0] DWs, the ferroelectric layers are separated by spacer layers, leading to negligible interlayer interaction. This behavior can be understood by a polar flat band and enables polarization switchability even within a half‐unit‐cell width [22]. Interestingly, recent experiments [28, 29] show that DWs formed between the *U* and *D_xy_* phases can adopt either high‐energy (HE) or low‐energy (LE) *Pbca* domain‐wall structures, depending on the atomic configuration at the domain‐wall center.

**FIGURE 2:**
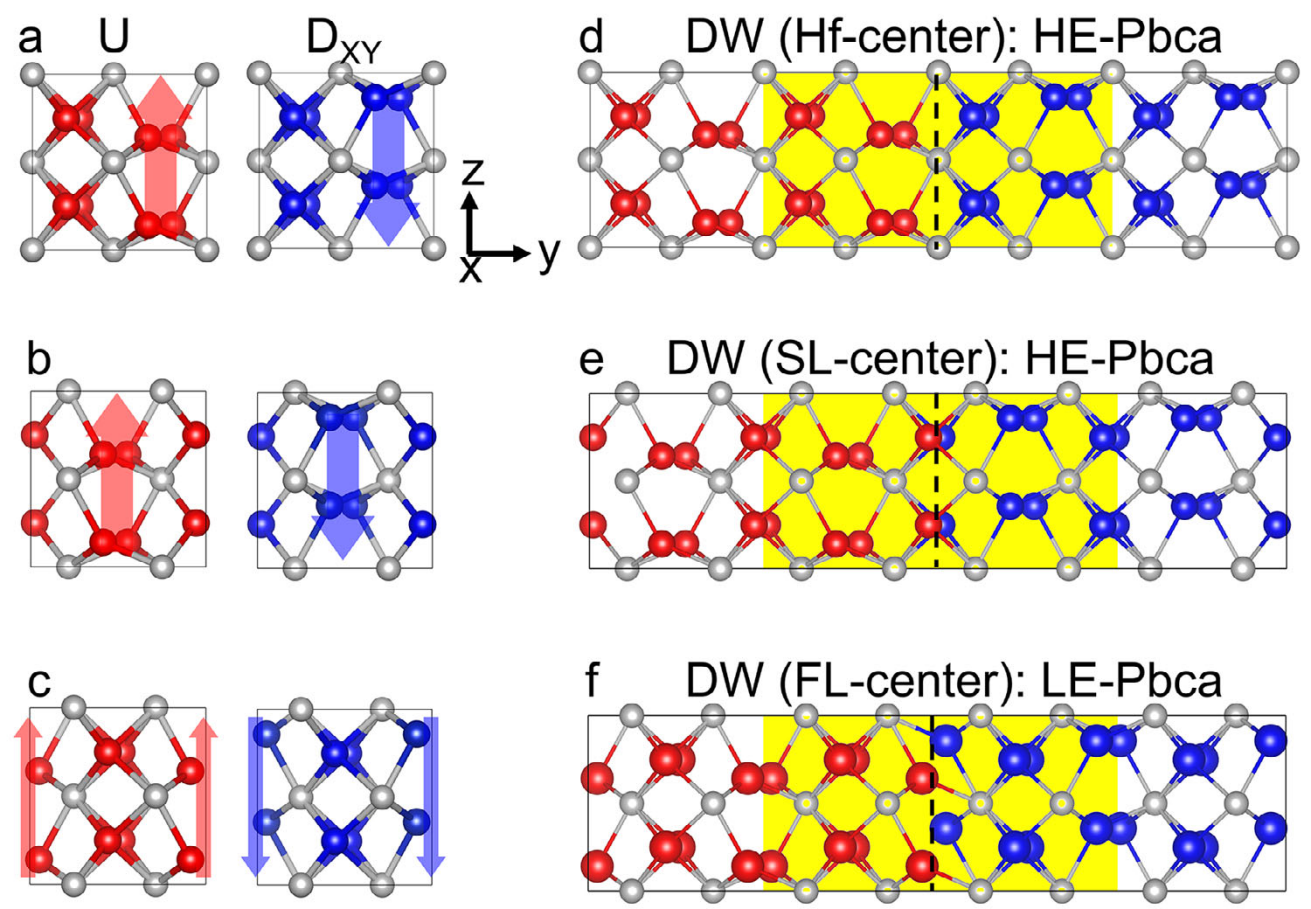
Crystallized DWs determined by DW‐centers along [0 1 0] direction. Atomic structures of the *U* and *D_xy_* phases are shown, where the Hf layer (a), spacer layer (SL) (b), and ferroelectric layer (FL) (c) are located at or near the faces perpendicular to the [0 1 0] direction of the unit cell, forming Hf‐centered (d), SL‐centered (e), and FL‐centered (f) DWs (dashed black lines), respectively. The blue and red arrows indicate the polarization directions in (a–c). The yellow shaded structures represent the high‐energy *Pbca* (HE‐*Pbca*) phase at Hf‐centered (d) and SL‐centered (e) DWs, and the low‐energy *Pbca* (LE‐*Pbca*) phase at FL‐centered DWs (f).

To gain structural and energetic insights into the *U*/*D_xy_* [0 1 0] DWs, we model *U*/*D_xy_* 180° DWs along the y‐direction, with the domain‐wall center located at the Hf (Figure 2a,d), spacer (Figure 2b,e) and ferroelectric (Figure 2c,f) layers. DWEs were estimated using first‐principles total energy calculations on a 1 × 8 × 1 supercell containing four up‐ and four down‐polarized unit cells (Figure S3a,c and e). The estimated DWE, using Equation S2, of −14.6 $mJ/m^{2}$ for Hf‐ and SL‐centered DWs is identical and negatively negligible, whereas the FL‐centered DW shows a positively negligible DWE of 8.7 $mJ/m^{2}$ (Figure S4), indicating their robust stability. We note that the DWEs are well converged with increasing supercell size (Table S1), confirming that the 1 × 8 × 1 supercell is sufficiently large to eliminate interactions between neighboring domain walls. Surprisingly, the up‐ and down‐polarization in adjacent unit cells across the Hf‐ (Figure S3b), SL‐ (Figure S3d), and FL‐centered (Figure S3f) DWs remain unsuppressed, confirming their sharp DW characteristics and consistency with earlier results [22, 33]. Notably, the Hf‐ and SL‐centered DW structures, containing oppositely polarized unit cells across the DW, adopt the antipolar HE‐*Pbca* phase (Figure 2d,e), whereas the FL‐centered configuration adopts the antipolar LE‐*Pbca* structure (Figure 2f). Which are in good agreement with previous experimental and theoretical results [28, 29].

To determine the atomic‐scale origin of the distinct DW structures specific to their wall centers, we analyze the eight phonon modes, $X_{2}^{\prime }$, $\Gamma_{15}^{z}$, $X_{5}^{y}$, $Y_{5}^{z}$, $Z_{5}^{x}$, 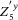, 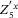, and $Y_{3}^{\prime }$ (Figure S5a) involved in the phase transition from the paraelectric cubic phase to the ferroelectric *Pca*2_1_ phase. Where $X_{2}^{\prime }$ is unstable, leads the transformation, while the rest of seven modes are stable (Figure S6). Interestingly, among these eight modes, only four $X_{2}^{\prime }$, $\Gamma_{15}^{z}$, $Y_{5}^{z}$ and $Z_{5}^{x}$ modes reverse their sign across the domain‐wall center in *U*/*D_xy_* DWs, while the rest of four modes remain unchanged (Figure S5b). Surprisingly, the band‐connected polar $\Gamma_{15}^{z}$ and antipolar $Y_{5}^{z}$ modes switch sign simultaneously, forming a polar mode pair that prevents any exchange between the spacer and ferroelectric layer positions, while the soft $X_{2}^{\prime }$ and stable $Z_{5}^{x}$ modes also switch sign simultaneously, constituting a soft mode pair. These phonon pairs have been used to explain the energetics and ferroelectric properties of the *U*/*D_xy_* Hf‐centered [0 1 0] DWs [33].

**FIGURE 3:**
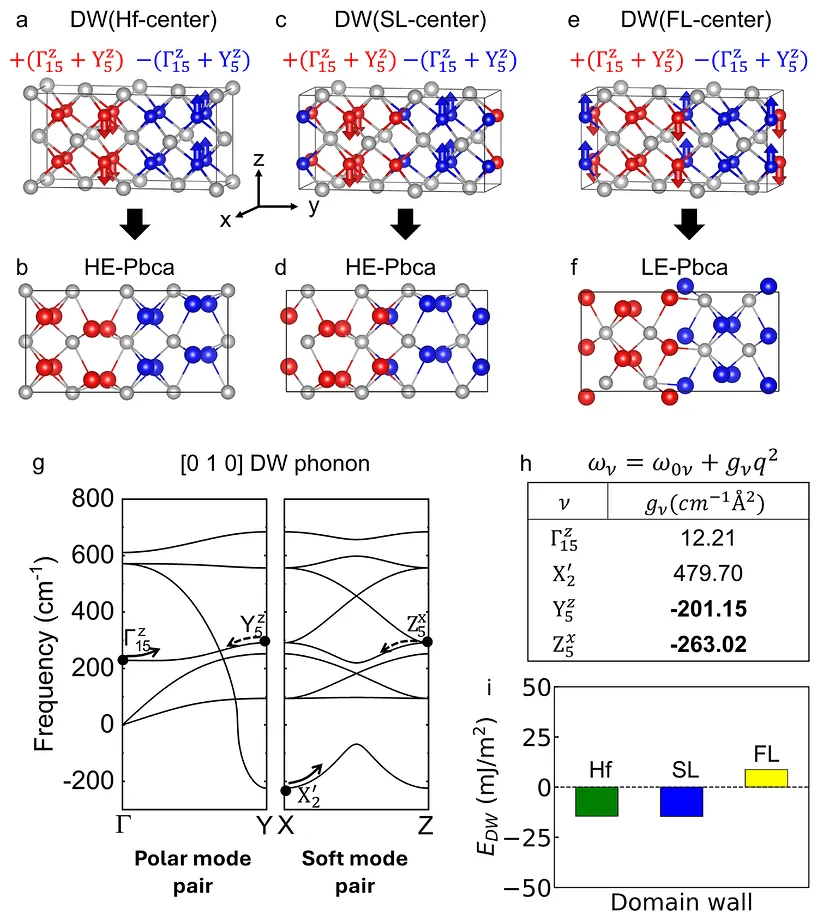
Atomic‐scale origin of distinct topologies and nearly zero DWEs of [0 1 0] DWs. Atomic displacements of pairing of the $\Gamma_{15}^{z}$ and $Y_{5}^{z}$ modes in a 24‐atom cubic unit cell are shown for the Hf‐centered (a), SL‐centered (b), and FL‐centered (c) *U*/*D_xy_* [0 1 0] DWs. In the Hf‐ and SL‐centered pairs, the front and back oxygen atoms within the ferroelectric layer displace in the same direction, whereas in the FL‐centered they displace in oppositely. The condensation of the Hf‐ and SL‐centered polar mode pairs leads to the transformation from cubic to HE‐*Pbca* (d, e), while FL‐centered transforms it into the LE‐*Pbca* phase (f). Phonon dispersion of the cubic phase along the [010] DW path (g) reveals the positive curvature for $\Gamma_{15}^{z}$ and $X_{2}^{\prime }$ modes (upward solid arrows), while negative curvatures for $Y_{5}^{z}$ and $Z_{5}^{x}$ modes (downward dashed arrows), and their estimated gradients by quadratic fitting (h). The DWEs estimated by total energy calculations (i).

Interestingly, we found that the oxygen displacements associated with the sign reversal of the $\Gamma_{15}^{z}$ and $Y_{5}^{z}$ modes, across the DWs strongly depend on the DW center. For the Hf‐ and SL‐centered DWs, all oxygen atoms within the ferroelectric layer displace in the same direction (Figure 3a,b), whereas for the FL‐centered DWs, the front and back oxygen atoms displace in opposite directions (Figure 3c) because they belong to oppositely polarized unit cells across the DW center. This difference is found to be the leading factor governing the distinct topologies of differently centered [0 1 0] DWs. Similarly, the oxygen displacements associated with the $X_{2}^{\prime }$ and $Z_{5}^{x}$ modes are identical for the Hf‐ and SL‐centered DWs (Figures S7b and S8b), while they differ for the FL‐centered DWs (Figure S9b). Since the $X_{5}^{y}$, 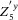, 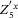 and $Y_{3}^{\prime }$ modes do not change, their signs are identical in Hf‐, SL‐ and FL‐centered DWs (Figures S7, S8 and S9). Surprisingly, the condensation of the polar and soft mode pairs along with the $X_{5}^{y}$, 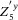, 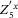, and $Y_{3}^{\prime }$ modes for the Hf‐ (Figure S7) and SL‐ (Figure S8) centered DWs transform the cubic reference phase into the HE‐*Pbca* (Figure 3b,d), whereas for the FL‐centered (Figure S9) DW it transforms it into the LE‐*Pbca* phase (Figure 3f).

Now, to gain insight into the nearly‐zero DWEs of all three *U*/*D_xy_* [0 1 0] DWs, we estimated the gradient energies associated with the band curvatures (Figure 3g) and gradient coefficient (*g*_ν_) (Figure 3h) of modes that reverse sign across the *U*/*D_xy_* DWs, which directly contribute to the DWEs [34]. Since the DW center does not affect gradient energy, it remains identical for all three Hf‐, SL‐, and FL‐centered DWs, and its estimated value (**using** Equation S3) of 28.9 *mJ*/*m* ^2^ is negligibly small. However, the DWEs obtained from total‐energy DFT calculations (Figures 3i and S4) are distinct for the SL‐ and FL‐centered DWs and are lower than the gradient‐energy estimate. This difference arises because the gradient energy is estimated analytically using identical phonon amplitudes of the equilibrium *Pca*2_1_ phase in each unit cell of the 1 × 8 × 1 supercell, whereas in the relaxed DW structures the amplitude of each mode varies slightly from its bulk value depending on its positive or negative band gradient coefficient (Figure 3h). Modes with negative curvature are enhanced, while those with positive curvature are slightly suppressed (Figures S10 and S11) near the DWs to reduce the energy of the DW configurations. Notably, this redistribution is weak in the FL‐centered DW (Figure S12), leading to only a minor reduction in its DWE, whereas it is stronger in the SL‐ and Hf‐centered DWs, yielding significantly lower, even slightly negative, DWEs.

### 2.3 Domain Walls Along [1 0 0] and [1 1 0] Crystallographic Directions

**FIGURE 4:**
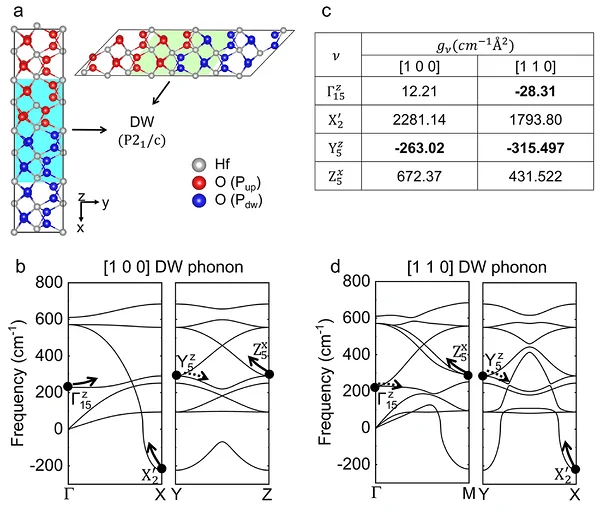
Crystallized DWs and moderate DWEs from counter‐cancelled band curvatures. (a) Atomic structures of *U*/*D_xy_* DWs along the [1 0 0] and [1 1 0] directions, where the *P*2_1_/*c* structures at DWs are shaded by cyan and light green boxes for [1 0 0] and [1 1 0] DWs, respectively. Phonon dispersions of the cubic reference phase along the [100] (b) and [110] (d) domain wall paths, along with the gradients of modes that reverse sign (c).

We now analyze the *U*/*D_xy_* DWs along the [1 0 0] and [1 1 0] directions (Figure 4a). The DWEs were estimated using the 8 × 1 × 1 supercell for the [1 0 0] DW (Figure S13a), whereas the [1 1 0] DW was modeled in a 1 × 8 × 1 supercell with diagonally tilted *U* and *D_xy_* unit cells (Figure S13c). Notably, in these DWs the DW center does not play a significant role, unlike in the [0 1 0] DWs. This is because, along the [1 0 0] and [1 1 0] directions, the Hf, spacer, and ferroelectric layers coincide at the DW center (Figure 4a). The moderately positive DWEs (estimated using Equation S2) of 310 $mJ/m^{2}$ and 190 $mJ/m^{2}$ for the [1 0 0] and [1 1 0] DWs, respectively, indicate their metastability, with the [1 1 0] DW energetically favored over the [1 0 0] DW. Notably, despite their metastable nature, up‐ and down‐polarization remains unsuppressed in adjacent unit cells across the DWs (Figure S13b,d), unlike in conventional ferroelectrics. Interestingly, structural analysis reveals that both DWs adopt the same low‐symmetry antipolar monoclinic *P*2_1_/*c* space group, each containing 24 atoms (Figure S14), while their structural parameters are distinct (Table S2).

Surprisingly, these *P*2_1_/*c* structures are energetically stabilized between the reference cubic and ferroelectric *Pca*2_1_ phases in bulk HfO_2_ (Table S2) and are distinct from the ground‐state monoclinic *P*2_1_/*c* phase, although they share the same space group. The *P*2_1_/*c* phase at the [1 0 0] DW, which has also been recently reported as a metastable state in bulk HfO_2_ [35], further strengthens our claim. To the best of our knowledge, the metastable *P*2_1_/*c* phase at the [1 1 0] DW has not been previously reported in HfO_2_ and is identified here for the first time. These low‐symmetry DW crystals prevent polarization suppression at the DWs despite positive DWEs, underscoring their critical role in ferroelectrics, and coexist with the polar phase in HfO_2_.

To understand the atomic‐scale origin of the DW structures along the [1 0 0] and [1 1 0] directions, we analyzed the atomic displacements of the $\Gamma_{15}^{z}$ and $Y_{5}^{z}$, $X_{2}^{\prime }$ and $Z_{5}^{x}$ modes across the DW centers of the [1 0 0] (Figure S15a,b) and [1 1 0] (Figure S16a,b) DWs. Surprisingly, we found that the condensation of the relevant modes for the [1 0 0] DWs, together with the non‐reversing modes (Figure S15) in the cell‐doubled cubic unit cell along the x‐direction, transforms it into an antipolar *P*2_1_/*c* phase (Figure S14a). Similarly, the condensation of the relevant modes for the [1 1 0] DWs, along with the non‐reversing modes in the diagonally tilted cell‐doubled cubic unit cell (Figure S16) associated with the [1 1 0] DWs, transforms the reference structure into a distinct antipolar *P*2_1_/*c* phase (Figure S14b). Thus, our results demonstrated that these two phases are not only stabilized at the DWs but also exist as metastable states in bulk HfO_2_. To reproduce the bulk structural and energetic properties of these phases, we have included their lattice vectors and atomic positions in Note S1. Moreover, their phonon dispersions (Figure S17c,d) reveal no imaginary frequencies, which confirms both phases are dynamically stable at low temperature.

Notably, despite having the same space groups, their DWEs of 310 $mJ/m^{2}$ and 190 $mJ/m^{2}$ (Figure S4) for the [1 0 0] and [1 1 0] DWs, respectively, are significantly different. To determine the origin of this difference, we estimated the gradient energies of both DWs. Since the wave vectors associated with [1 0 0] and [1 1 0] DWs differ, the band curvatures of the $\Gamma_{15}^{z}$, $X_{2}^{\prime }$, $Y_{5}^{z}$ and $Z_{5}^{x}$ modes are also different (Figure 4b,d), resulting in different gradient energies for the same phonon modes along the [1 0 0] and [1 1 0] DWs (Figure 4c). As a result, the gradient energies, using Equation S3, for the [1 0 0] and [1 1 0] DWs are 508.2 $mJ/m^{2}$ and 351.7 $mJ/m^{2}$ (Figure S2), respectively, indicating a substantial difference between the two, consistent with the trend in DWEs obtained from DFT total‐energy calculations. However, the DWEs for both [1 0 0] and [1 1 0] DWs calculated from total energies are significantly lower than the analytically estimated gradient energies. This discrepancy arises because, in the relaxed DW structures, the amplitudes of modes with negative curvature are enhanced, whereas those with positive curvature are reduced (Figures S18 and S19), thereby lowering the total energy of the DW configurations, similar to what occurs in [0 1 0] DWs.

### 2.4 Domain Walls Along Arbitrary [m n 0] Crystallographic Direction

After establishing a comprehensive understanding of DWs along the well‐defined [1 0 0], [0 1 0], and [1 1 0] crystallographic directions, we generalize our analysis to in‐plane DWs along arbitrary [m n 0] directions. We find that DWs with orientations between [1 0 0] and [1 1 0] adopt structures composed of coexisting [1 0 0] and [1 1 0] DW segments, with proportions determined by the wall orientation, and the corresponding DWE can be expressed as a linear combination of the DWEs of the [1 0 0] and [1 1 0] DWs. Similarly, for DW orientations between [1 1 0] and [0 1 0], the DW structures consist of coexisting [0 1 0] and [1 1 0] segments in their respective proportions, and the corresponding DWE follows a linear combination of the [0 1 0] and [1 1 0] DW energies. Thus, by knowing the DWEs of the [1 0 0]‐, [1 1 0]‐, and [0 1 0]‐DWs, one can predict the DWE of any arbitrary [m n 0] DWs, as follows,

(1) $$E_{DW}^{\left[m\;n\;0\right]}=\left\{\begin{array}{c} \left(1-2x\right)E_{DW}^{\left[010\right]}+2xE_{DW}^{\left[110\right]},\;\left(0\leq x\leq 0.5\right)\\ \left(2-2x\right)E_{DW}^{\left[110\right]}+\left(2x-1\right)E_{DW}^{\left[100\right]},\;\left(0.5\leq x\leq 1\right) \end{array}\right.\;$$

where, $E_{DW}^{\left[mn0\right]}$ is the DWE of [m n 0] direction DWs. $E_{DW}^{\left[010\right]}$, $E_{DW}^{\left[100\right]}$, $E_{DW}^{\left[110\right]}$ are DWEs of *U*/*D_xy_* DWs along the [0 1 0], [1 0 0], and [1 1 0] directions. *x* is the mixing parameter that determines the relative contributions of the reference [1 0 0], [0 1 0], and [1 1 0] DW energies to the DWE of an arbitrary DW orientation. We define it as *x*  =  2/πtan ^−1^(*m*/*n*) which accurately predict the mixing contributions of the [1 0 0], [0 1 0], and [1 1 0] DW components for arbitrary crystallographic orientations.

**FIGURE 5:**
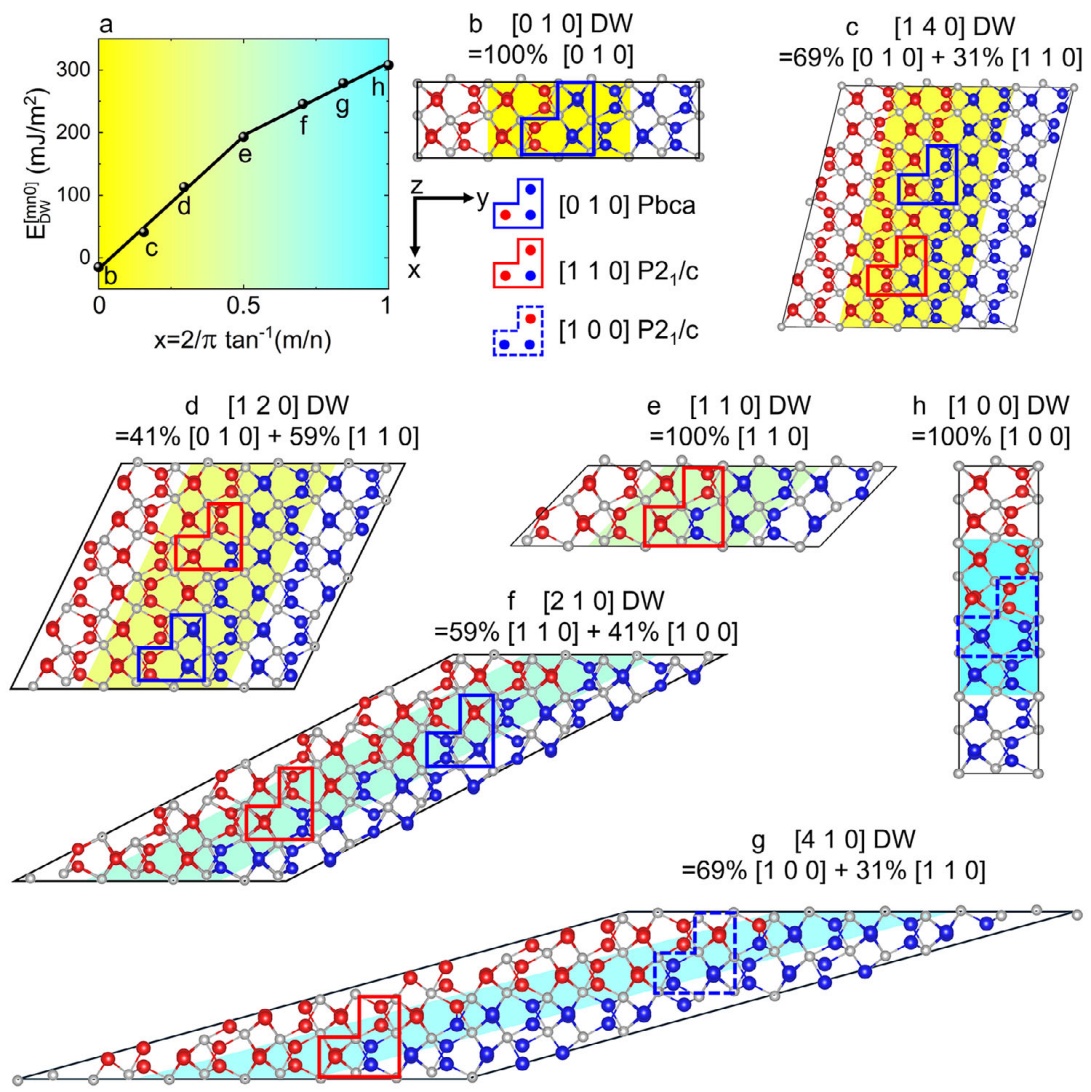
Predictable energies and structures of crystallized DWs along arbitrary crystallographic directions. (a) DWEs along arbitrary [m n 0] directions; solid circles and line represent estimated and predicted values, respectively. Optimized atomic structures of DWs are shown for [0 1 0] (b), [1 4 0] (c), [1 2 0] (d), [1 1 0] (e), [2 1 0] (f), [4 1 0] (g), and [1 0 0] (h). The shaded regions with different colors at DWs depict the crystallized DW structures. The crystal structure of the [0 1 0] DW is a perfect *Pbca* (b), whereas the [1 1 0] (e) and [1 0 0] (h) DWs are perfect *P*2_1_/*c* phases. The DW structures along the intermediate directions between [0 1 0] and [1 1 0], such as [1 4 0] and [1 2 0], contain components of the [0 1 0] *Pbca* and [1 1 0] *P*2_1_/*c* Phases in proportions determined by their directional ratios. Similarly, the DW structures along the intermediate directions between [1 1 0] and [1 0 0], such as [2 1 0] and [4 1 0], contain components of the [1 1 0] *P*2_1_/*c* and [1 0 0] *P*2_1_/*c* phases. The smallest structural units used to identify the [0 1 0] *Pbca*, [1 1 0] *P*2_1_/*c*, and [1 0 0] *P*2_1_/*c* phases at the DWs are indicated by solid blue, solid red, and dashed blue L‐shaped regions, respectively. Red and blue dots denote oxygen from up and down‐polarized unit cells, respectively.

To verify the DWE predictability of our theoretical framework, we first calculated the DWEs of the [1 4 0], [1 2 0], [2 1 0], and [4 1 0] DWs (Figure 5) using Equation S2 with total energies obtained from first‐principles simulations. Remarkably, the estimated DWEs (Figure 5a) are in great agreement with those predicted by Equation (1), confirming the predictive capability of our mechanism. Notably, the linearity of the DWEs suggests negligible interaction among polar and antipolar phases, making this a suitable feature for targeted memory coding in ferroelectrics, which is unlikely in conventional ferroelectrics.

Furthermore, the DW structures along arbitrary crystallographic directions can be decomposed into the crystal structures of the [1 0 0] *Pbca*, [0 1 0] *P*2_1_/*c*, and [1 1 0] *P*2_1_/*c* perfect crystallized structures of [1 0 0], [0 1 0], and [1 1 0] DWs, respectively. The structural components of these phases can be directly estimated from the value of *x* for the arbitrary DWs, following the same linear dependence of Equation (1) as,

(2) $$\begin{array}{ccc} & & S_{DW}^{\left[m\;n\;0\right]}=\\ & & \left\{\begin{array}{c} \left(1-2x\right)\;\left[0\;1\;0\right]Pbca+2x\;\left[1\;1\;0\right]\;P2_{1}/c,\\ \;\left(0\leq x\leq 0.5\right)\\ \left(2-2x\right)\;\left[1\;1\;0\right]P2_{1}/c+\left(2x-1\right)\;\left[1\;0\;0\right]\;P2_{1}/c,\\ \;\left(0.5\leq x\leq 1\right) \end{array}\;\times 100\%\right. \end{array}$$

where $S_{\mathrm{DW}}^{\left[m\;n\;0\right]}$ is the structure of the arbitrary [*m* *n* 0] DW. Using Equation (2), we estimated that the [1 4 0] DW consists of 69% [0 1 0] *Pbca* and 31% [1 1 0] *P*2_1_/*c* phases, while the [1 2 0] DW consists of 41% [0 1 0] *Pbca* and 59% [1 1 0] *P*2_1_/*c* phases (Figure 5b–e). Similarly, we found that the [2 1 0] (Figure 5f) and [4 1 0] (Figure 5g) DWs, which lie between the [1 1 0] (Figure 5e) and [1 0 0] (Figure 5h) DWs, consist of 59% [1 1 0] *P*2_1_/*c* + 41% [1 0 0] *P*2_1_/*c* and 69% [1 1 0] *P*2_1_/*c* + 31% [1 0 0] *P*2_1_/*c* components, respectively.

To further verify the structural predictability of our scheme, we manually count the approximate units of [0 1 0] *Pbca* (Figures 5b and S20a) and [1 1 0] *P*2_1_/*c* (Figures 5e and S20b) phases in the DW structures in [1 4 0] (Figures 5c and S20c) and [1 2 0] (Figures 5d and S20d) DWs. For the [1 4 0] DW, we identified five and a half units of the [0 1 0] *Pbca* phase and two units of the [1 1 0] *P*2_1_/*c* phase (Figure S20c), corresponding to approximately 73% and 27% components, respectively. Similarly, the [1 2 0] DW contains three units of the [0 1 0] *Pbca* phase and two and a half units of the [1 1 0] *P*2_1_/*c* phase (Figure S20d), corresponding to approximately 55% and 45% contributions, respectively. These values are in good agreement with the phase fractions estimated from Equation (2). Thus, our findings demonstrate that the structural and energetic properties of the perfectly crystallized [1 0 0], [1 1 0], and [0 1 0] DWs can serve as descriptors for analyzing DWs along arbitrary [*m* *n* 0]directions in the *xy*‐plane, as they can be readily obtained from first‐principles calculations.

### 2.5 Enabling Chessboard‐Type Ultra‐High‐Density Memories

**FIGURE 6:**
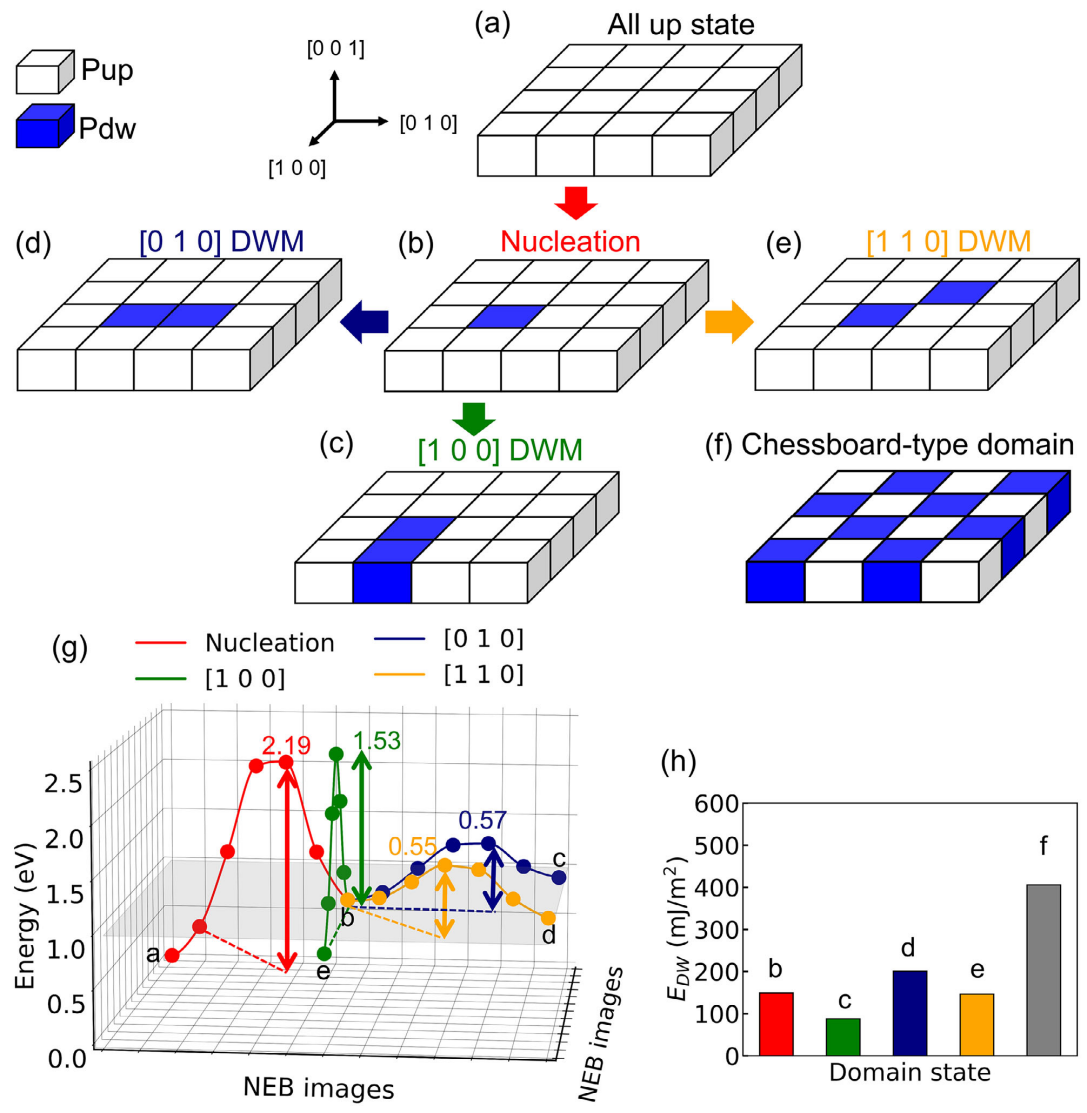
Stabilization of chessboard‐type ultrasmall domains and their mobility. Schematics of the all‐up‐polarized state (a), nucleation state with one down‐polarized unit cell (b), and adjacent down‐polarized unit cell along [1 0 0] (c), [0 1 0] (d), and [1 1 0] (e). (f) Schematic of a chessboard‐type ferroelectric domain architecture, where individual unit‐cell‐scale domains are controlled by an external electric field. (g) Estimated energy barriers separating distinct domain wall configurations. (h) Domain wall energies for each domain configuration.

Building upon the discussion in the preceding sections regarding stably localized DWs and the resultant up‐and‐down polarized domains, here we extend their potential implications and integration into device applications. We analyze the creation and annihilation of domains by constructing an up‐polarized monodomain configuration in a 4 × 4 × 1 supercell (Figure 6a) and flipping the polarization in a single unit cell (Figure 6b). Surprisingly, the single flipped state exhibits remarkable stability, yielding a DWE of 149.5 *mJ*/*m* ^2^ (Figure 6h) and retaining nearly bulk‐like polarization (Figure S21b). The monodomain state and nucleated state with one flipped dipole are separated by a significantly high energy barrier (Figure 6g), suggesting that once nucleated, the system is unlikely to revert to the monodomain state. When the polarization is flipped in adjacent unit cells along the [1 0 0] (Figure 6c), [0 1 0] (Figure 6d), and [110] (Figure 6e) directions, the estimated DWEs of 87.8 *mJ*/*m* ^2^, 200.4 *mJ*/*m* ^2^, and 146 *mJ*/*m* ^2^, respectively, remain relatively small (Figure 6h), indicating the metastability of these domains.

Notably, each unit cell with reversed polarization retains nearly bulk‐like polarization (Figure S21), while the P_x_ (Figure S22) and P_y_ (Figure S23) components of the polarization remain nearly zero, confirming the robustness of the ferroelectric state. Importantly, these respective configurations are separated by sizable barriers of 0.57, 1.53, and 0.55 eV (Figure 6g), indicating that thermal fluctuations alone cannot switch the polarization, enabling long‐term retention of domain states. We note that the polarization switching barrier may vary for unconventional switching pathways [32]. In our nudged elastic band (NEB) calculations, we considered a switching pathway in which only the oxygen atoms in the ferroelectric layers are displaced between the Hf layers. Interestingly, at the transition state, only the unit cell undergoing polarization switching exhibits suppressed polarization, whereas the neighboring unit cells retain substantial polarization (Figure S24). This indicates that the stability of the neighboring unit cells is largely preserved during the switching process.

Furthermore, we modeled a chessboard‐type DW configuration in which each unit cell is surrounded by oppositely polarized neighbors (Figure S25a). Surprisingly, this configuration exhibits remarkable stability, yielding a DWE of 405.8 *mJ*/*m* ^2^ (Figure 6h) while maintaining nearly bulk‐like polarization in each unit cell (Figure S25b), while the P_x_ (Figure S25c) and P_y_ (Figure S25d) components of the polarization are zero. This represents the narrowest square‐shaped domains (Figure 6f), in which each unit cell can switch its polarization independently, enabling a chessboard‐like ferroelectric domain architecture where the polarization of each unit cell can be precisely switched without affecting the ferroelectric states of neighboring unit cells. With an in‐plane unit‐cell area of 27 Å^2^, this design enables ferroelectric domain densities up to ∼400 *Tbit*/*cm* ^2^, a record among known ferroelectrics and near the physical limit of the ferroelectric material. We note that the estimated memory density of ∼400 *Tbit*/*cm* ^2^ represents the ultimate theoretical limit of the achievable memory density. Since HfO_2_ is compatible with CMOS technology, these stable chessboard‐type domains are well suited for integration into large‐scale two‐dimensional crossbar arrays with ultranarrow feature sizes in ferroelectric field‐effect transistors (FeFETs), enabling synaptic functionalities for neuromorphic computing [36, 37, 38] and beyond.

## 3 Conclusion

In summary, we identify ferroelectric DW crystals formed by 180° domain walls along arbitrary crystallographic directions, which adopt well‐defined low‐symmetry structures with low energies. We establish that the DWEs and structures for arbitrary directions can be reliably approximated as linear combinations of the [0 1 0], [1 0 0], and [1 1 0] DWs, which are readily accessible from first‐principles simulations and serve as robust descriptors for predicting energetics in arbitrary directions. Remarkably, these DW crystals prevent polarization suppression near domain walls, retaining bulk‐like polarization in each domain regardless of direction and size. Each domain is separated by substantial energy barriers (0.55–2.3 eV), ensuring stability against thermal fluctuations and long‐term data retention. We further demonstrate that polarization switching can occur within a single unit cell while retaining bulk‐like polarization and high stability, potentially enabling chessboard‐type ultra‐high‐density of ferroelectric domains up to ∼400 *Tbit*/*cm* ^2^. Importantly, our mechanism reveals the microscopic origins of experimentally observed DW structures and predicts new ones, demonstrating strong predictive power. Thus provide a comprehensive understanding of DW structures and dynamics in HfO_2_ and enables the design of domain structures for improved ferroelectric memories.

## 4 Methods

### 4.1 Computational Details

The first‐principles simulations within this work were performed using density functional theory (DFT) within a plane‐wave pseudopotential framework as implemented in the Vienna Ab initio Simulation Package (VASP) [39, 40, 41]. The projector augmented‐wave (PAW) [42] method was employed with the generalized gradient approximation (GGA) and the PBEsol [43] functional for the exchange‐correlation energy. The valence electronic configurations considered 5*s* ^2^5*p* ^6^6*s* ^2^5*d* ^2^ for Hf and 2*s* ^2^2*p* ^4^ for O atoms. The plane‐wave energy cutoff was set to 500 *eV*. For Brillouin zone integrations, Monkhorst–Pack (MP) [44] k‐point meshes of 8 × 8 × 8 was used for the single unit cell of *Pca*2_1_ phase of HfO_2_. While 8 × 1 × 8, 1 × 8 × 8 and 1 × 1 × 8 for [0 1 0], [1, 0, 0] and [1 1 0] DWs, respectively. Structural optimizations were performed until the total energy converged to within 10^−^^7^ *eV* and the residual forces were below 10^−3^ *eV*/Å. Phonon dispersions of primitive unit cell of cubic phase of HfO_2_ were obtained using density functional perturbation theory (DFPT) as implemented in VASP.

## Author Contributions

**Pawan Kumar**: conceptualization, investigation, writing – original draft, methodology, validation, visualization, writing – review and editing, formal analysis, project administration, data curation. **Hyun‐Jae Lee**: conceptualization, investigation, writing – original draft, funding acquisition, methodology, validation, writing – review and editing, data curation. **Yungyeom Kim**: data curation, formal analysis, methodology. **Jihoon Choi**: data curation, investigation. **Jun Hee Lee**: conceptualization, funding acquisition, validation, writing – review and editing, supervision, resources.

## Funding

This work was supported by the Nano & Material Technology Development Program (Grants No. RS‐2024‐00404361, RS‐2024‐00444182), No. RS‐2025‐24535610, and No. RS‐2023‐ 00257666 through the National Research Foundation of Korea (NRF) funded by the Korean government (Ministry of Science and ICT). This work was also supported by Industrial Technology Innovation Program (Grant No. RS2025‐06642983), the Korea Institute for Advancement of Technology (KIAT) grant funded by the Korea Government (MOTIE) (Grant No. P0023703, HRD Program for Industrial Innovation), and the National Supercomputing Center at the Korea Institute of Science and Technology Information (KISTI) with supercomputing resources including technical support (Grants No. KSC‐2023‐CRE‐0547, and No. KSC‐2024‐ CRE‐0545). HJL acknowledges that this work was supported by the National Research Foundation of Korea (NRF) grant funded by the Korean government (MSIT) (RS‐2025‐00517117). National Supercomputing Center with supercomputing resources including technical support (Grant No. KSC‐2025‐CRE‐0234).

## Conflicts of Interest

The authors declare no conflicts of interest.

## Supporting information




**Supporting File**: advs77599‐sup‐0001‐SuppMat.pdf.

## Data Availability

The data that support the findings of this study are available on request from the corresponding author. The data are not publicly available due to privacy or ethical restrictions.
