# Supplementary material for: Crystallized Domain Walls in HfO2 Enable Chessboard‐Type Ultra‐High‐Dense Ferroelectric Memory
Source: Adv Sci (Weinh). 2026 Sep 13:e77599. Online ahead of print. doi: 10.1002/advs.77599 (PMC13572862; doi:10.1002/advs.77599)
Supplement: Supplementary file 1 [file ADVS-9999-e77599-s001.pdf]

Supporting Information

Supporting Information is available from the Wiley Online Library or from the author.

In general, DWs in ferroelectrics stabilize within a widely spread transition region between two oppositely polarized domains, which severely limits the achievable memory density. Here, the reported DWs in HfO_2 constitute perfect crystalline structures along arbitrary crystallographic directions, enabling the formation of the narrowest square-shaped chessboard-type domains. This unique characteristic could enable ultra-high-density memory with capacities up to ~400 $Tbit/cm^2$.

TOC figure Title: **Ultra-small square sub-nm² ferroelectric domains enabled by crystallized domain walls**

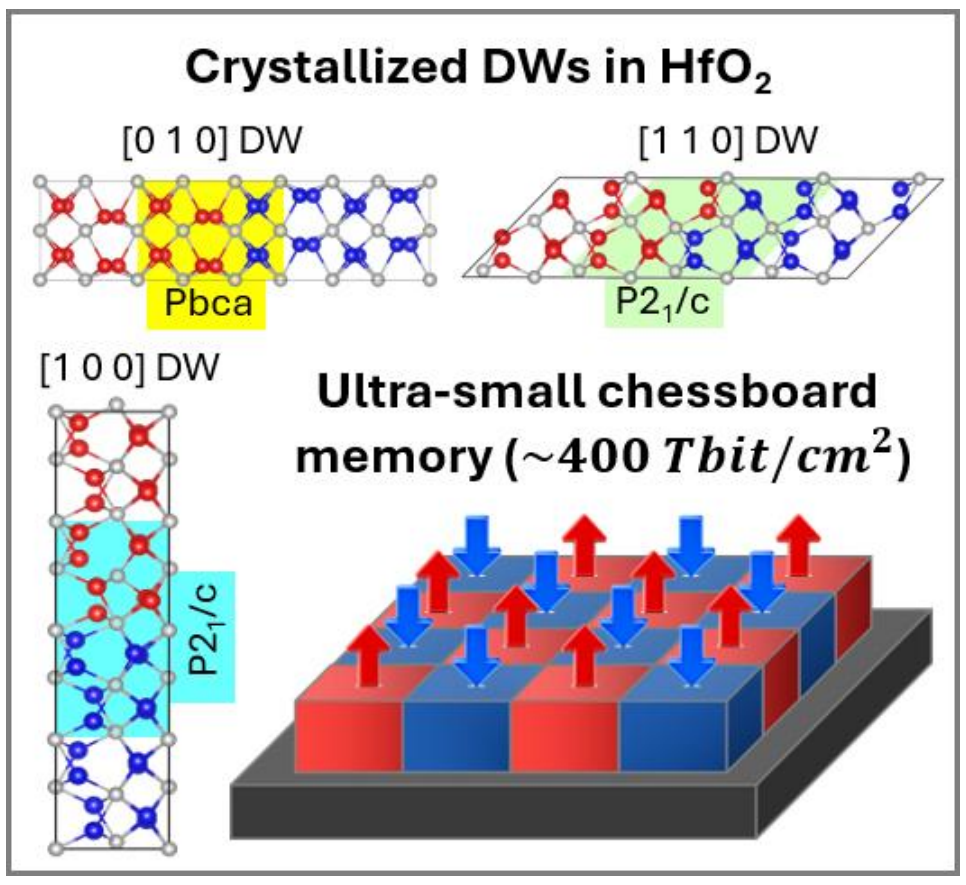

ToC figure ((Please choose one size: 55 mm broad × 50 mm high or 110 mm broad × 20 mm high. Please do not use any other dimensions))

Supporting Information

Crystallized domain walls in HfO_2 enable chessboard-type ultra-high-dense ferroelectric memory

Pawan Kumar[†], *Hyun-Jae Lee*[†], *Yungyeom Kim*[†], *Jihoon Choi and Jun Hee Lee*[*]

E-mail: junhee@unist.ac.kr
†These authors equally contributed to this work.

Estimation of polarization

The polarization in different ferroelectric phases was obtained by multiplying the Born effective charges (Z^*) of each atom with its displacements (d) from the centrosymmetric reference phase as follows,

$$P_s = \frac{e}{V}\sum_i Z_i^* . d_i, \qquad \text{(S1)}$$

where e and V represent the electronic charge and supercell volume, respectively, while i denotes the atomic site in the supercell.

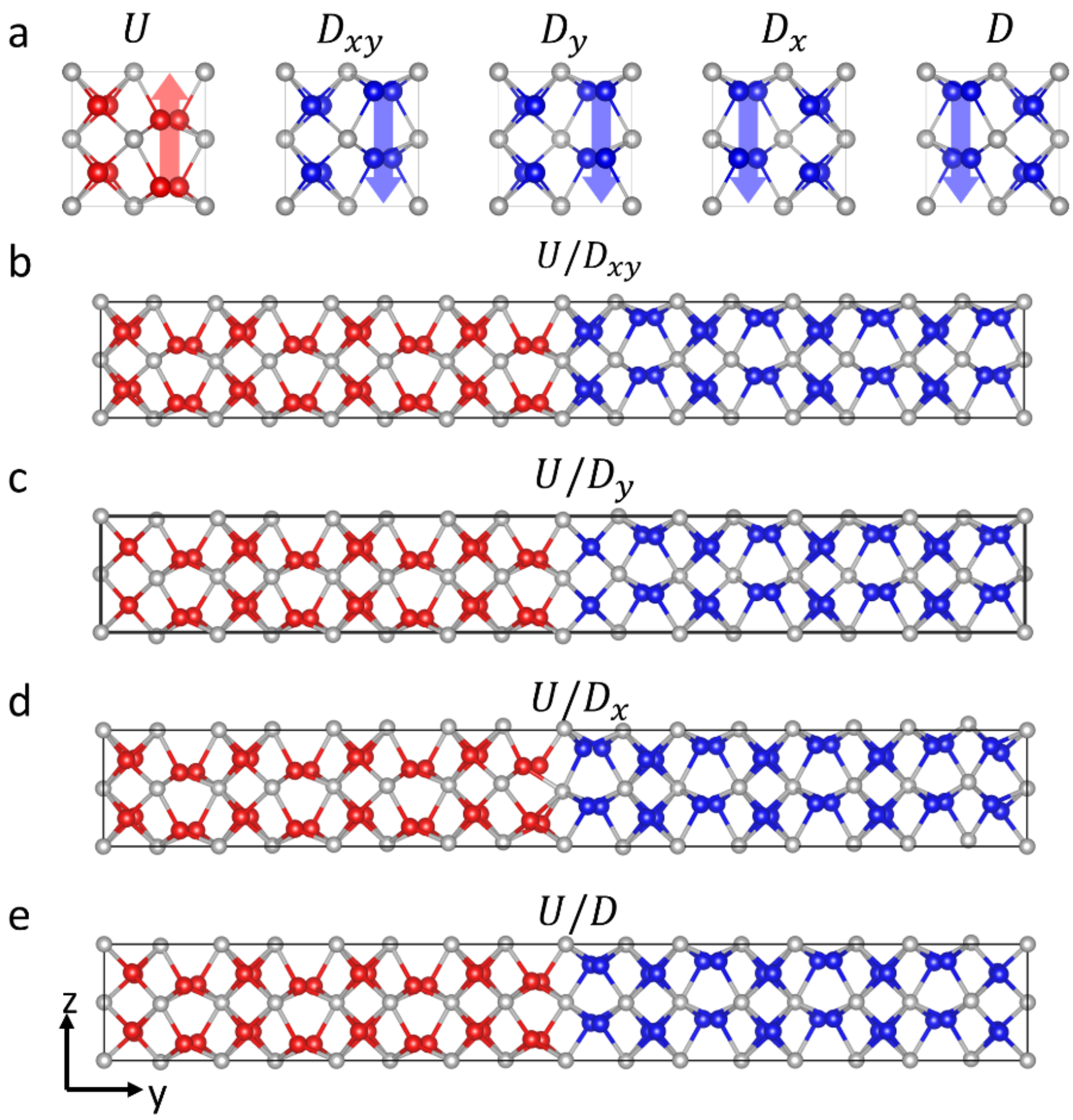

Figure S1. Atomic structures of up-polarized (U) and distinct symmetry equivalent down-polarized (D_{xy}, D_y, D_x and D) $Pca2_1$ phases (a), and their domain walls configurations along [0 1 0] direction in 1x8x1 supercell (b, c, d, and e).

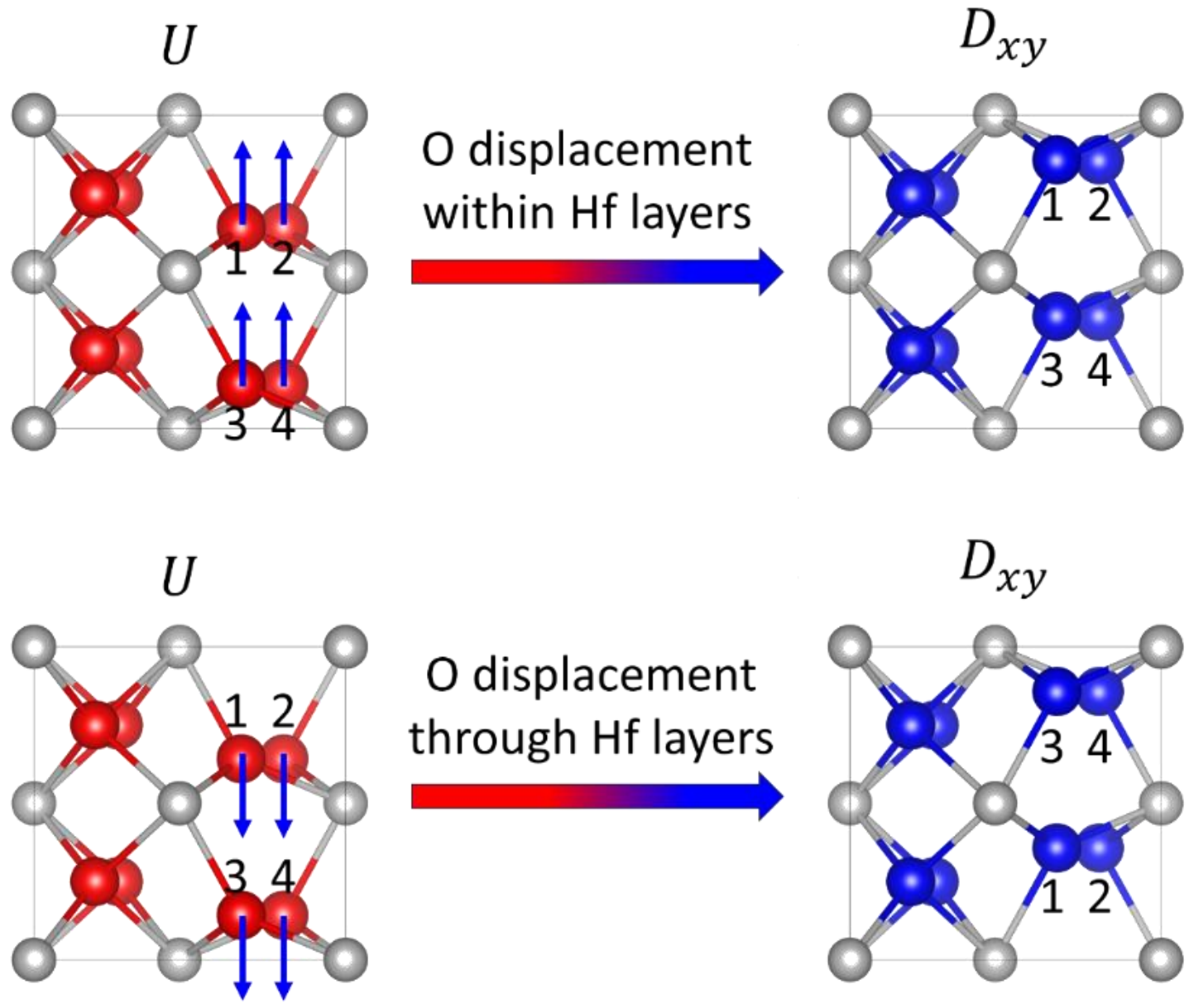

Figure S2. Polarization switching pathways between the U and D_{xy} phases, showing oxygen displacement within Hf layers (top) and through Hf layers (bottom).

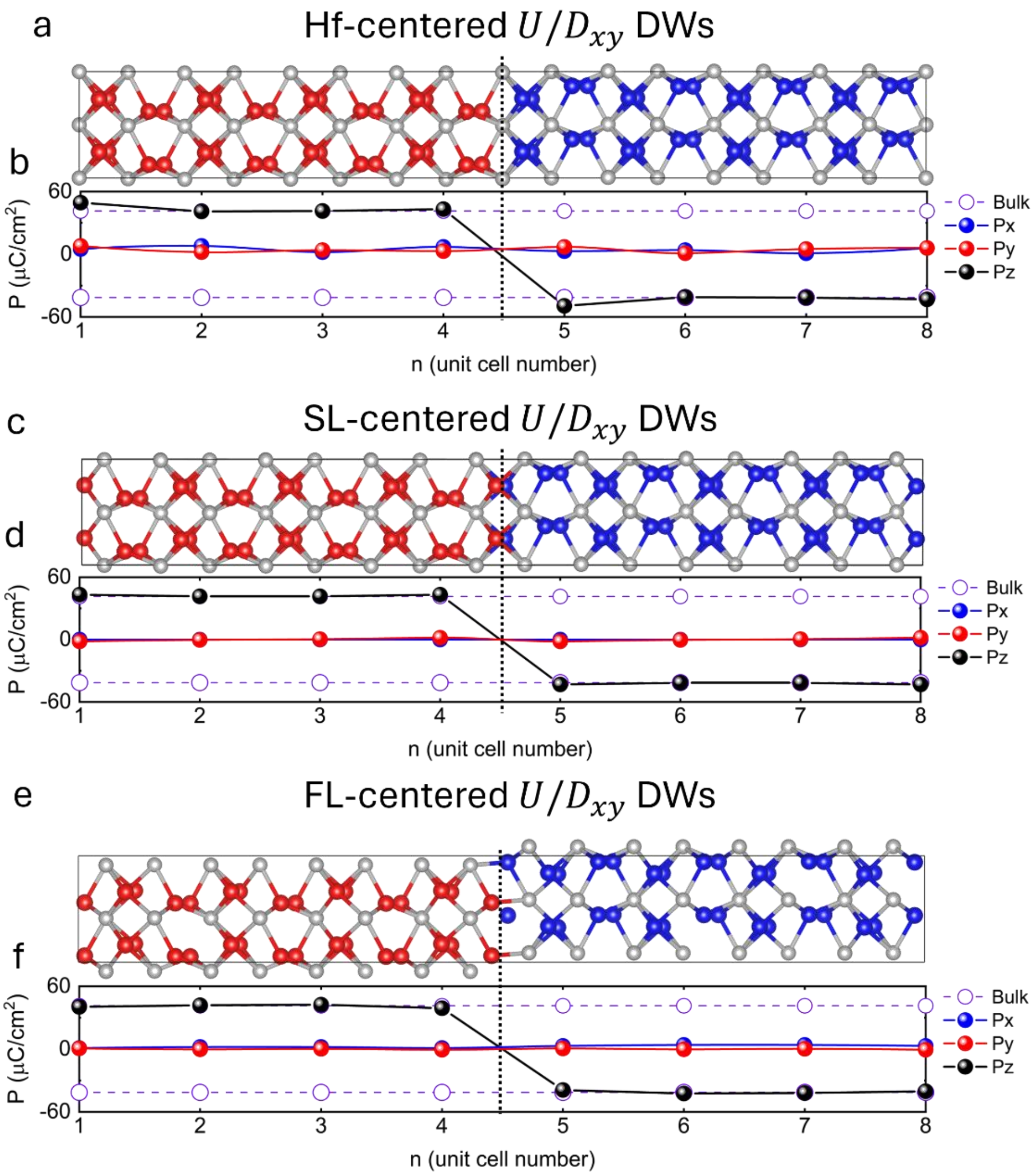

Figure S3. Atomic structures and the corresponding polarization in each unit cell of a 1×8×1 supercell for Hf-centered (a, b), SL-centered (c, d), and FL-centered (e, f) U/D_{xy} domain walls along the [010] direction.

Estimation of Domain wall energy

To assess the stability of DWs, we evaluate the domain wall energy (DWE), which can be directly obtained from density functional theory (DFT) calculations such as[1],

$$E_{DW}^{DFT} = \frac{E_{DW}-E_{MD}}{2A}, \quad \text{(S2)}$$

where E_{DW}^{DFT} is the domain wall energy. E_{DW} and E_{MD} are the total energies of DW and mono-domain structures, respectively, computed within DFT simulations, and A is the cross-sectional area of the DW configuration. The factor of two in the denominator accounts for the two domain walls in the periodic supercell.

Table S1. Estimated DWEs of the [100] and Hf-centered [010] U/D_{xy} DWs as a function of supercell size, demonstrating the convergence of the DWE with respect to DW configurations size of $2U/2D_{xy}$ (four-unit cell), $3U/3D_{xy}$ (six-unit cell) and $4U/4D_{xy}$ (eight-unit cell) supercells.

DW supercell	$E_{DW}^{[010]}$ (mJ/m^2)	$E_{DW}^{[100]}$ (mJ/m^2)
$2U/2D_{xy}$	-13.4	308
$3U/3D_{xy}$	-13.8	309
$4U/4D_{xy}$	-14.6	310

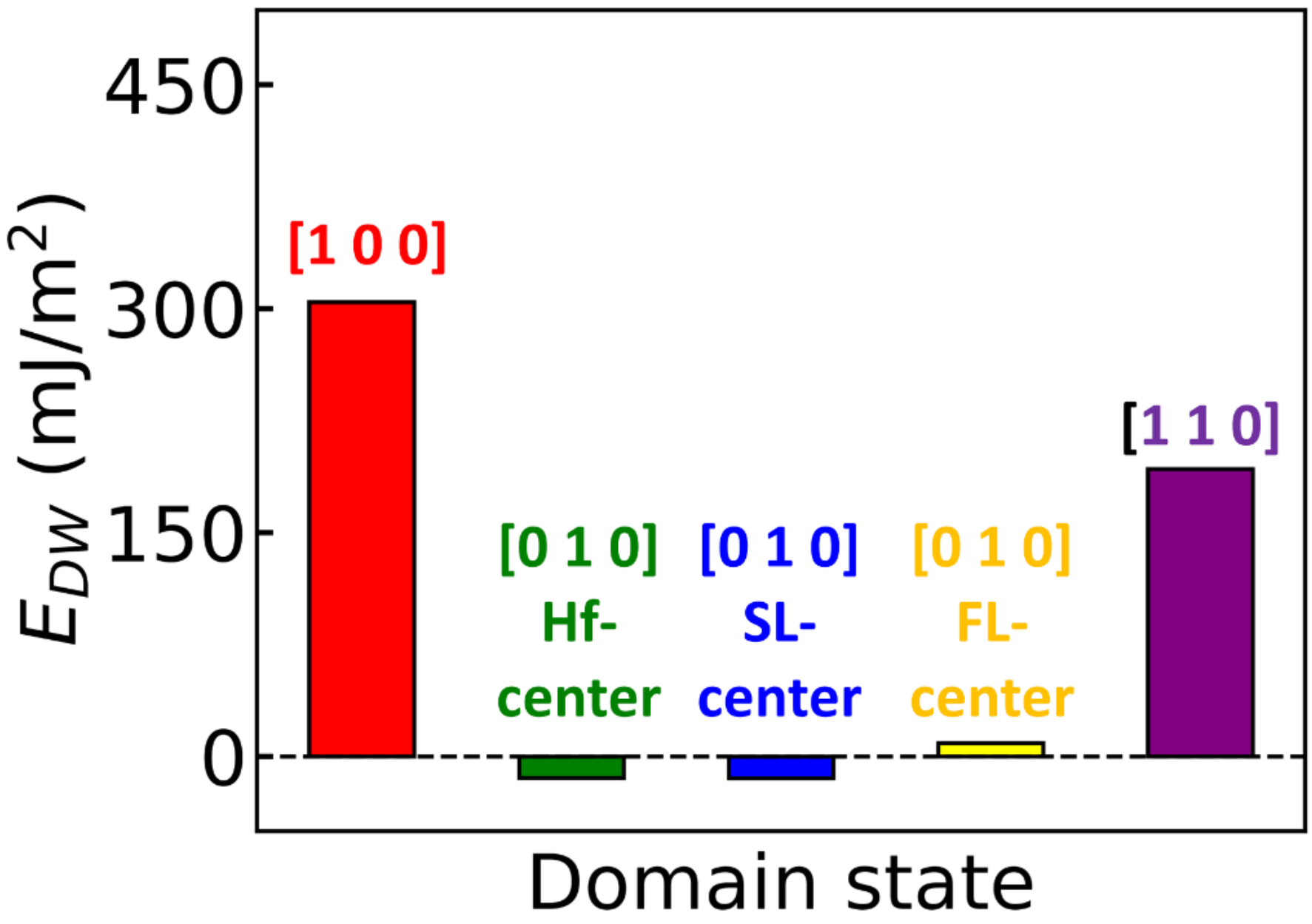

Figure S4. Estimated domain wall energy (E_{DW}), calculated using Equation S2, along the [100], [010], and [110] crystallographic directions. The [010] direction exhibits three distinct DW types as Hf-centered, SL-centered, and FL-centered.

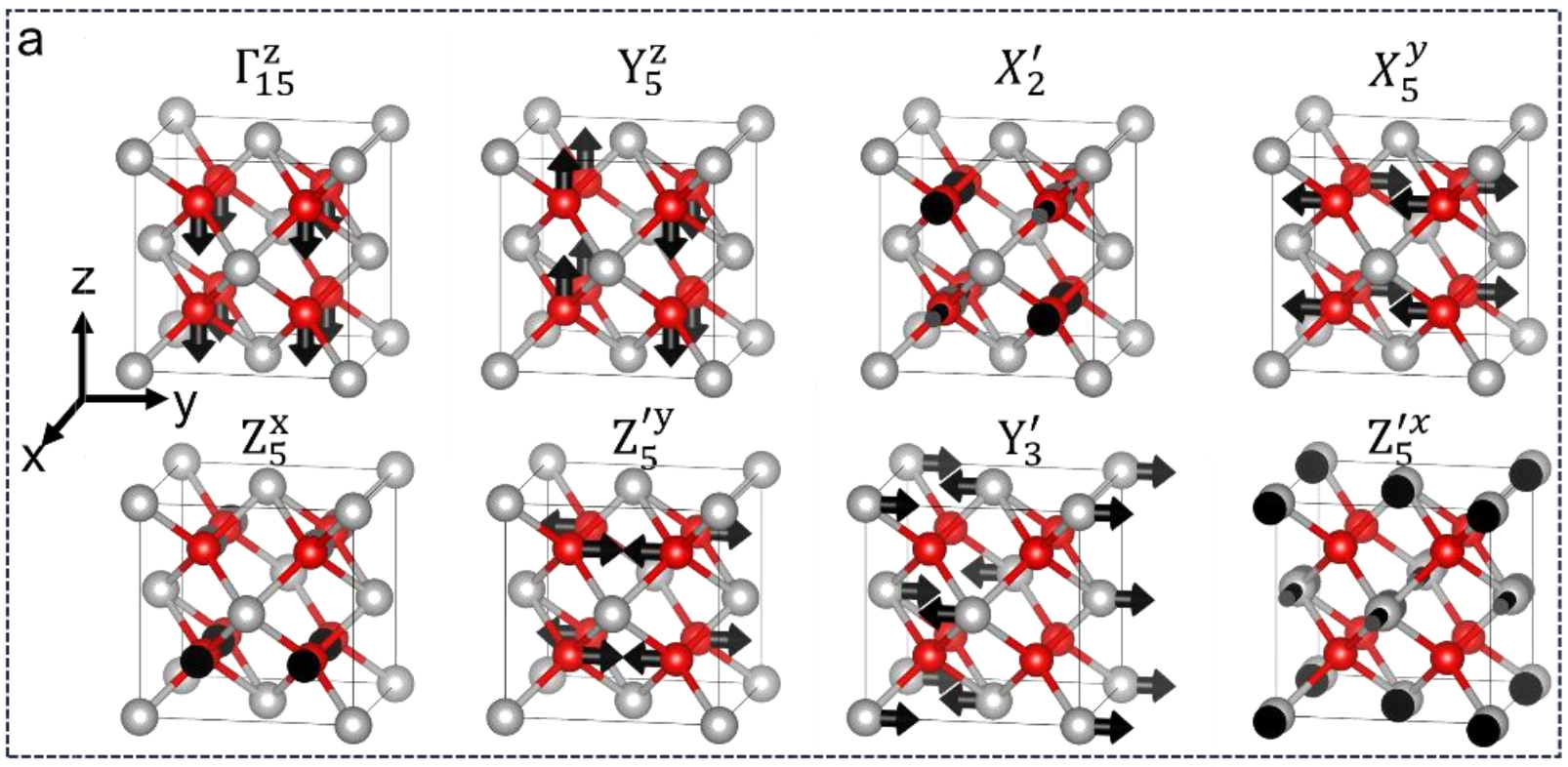

b

Structure	Γ^{z}_{15}	X'_{2}	X^{y}_{5}	Y^{z}_{5}	Z^{x}_{5}	Z'^{y}_{5}	Z'^{x}_{5}	Y'_{3}
U	0.410	0.501	0.389	0.390	0.370	0.114	0.165	0.087
Dxy	-	-	+	-	-	+	+	+
D	-	+	-	+	-	+	+	-
Dx	-	-	+	+	+	-	-	-
Dy	-	+	-	-	+	-	-	+

Figure S5. (a) Atomic displacement visualization of phonon modes of the cubic reference phase that transform it into the $Pca2_1$ phase. (b) Corresponding mode amplitudes in the up- and down-polarized states, where "+" and "–" indicate non-reversal and reversal of modes, respectively, in the down-polarized phase relative to the up-polarized phase.

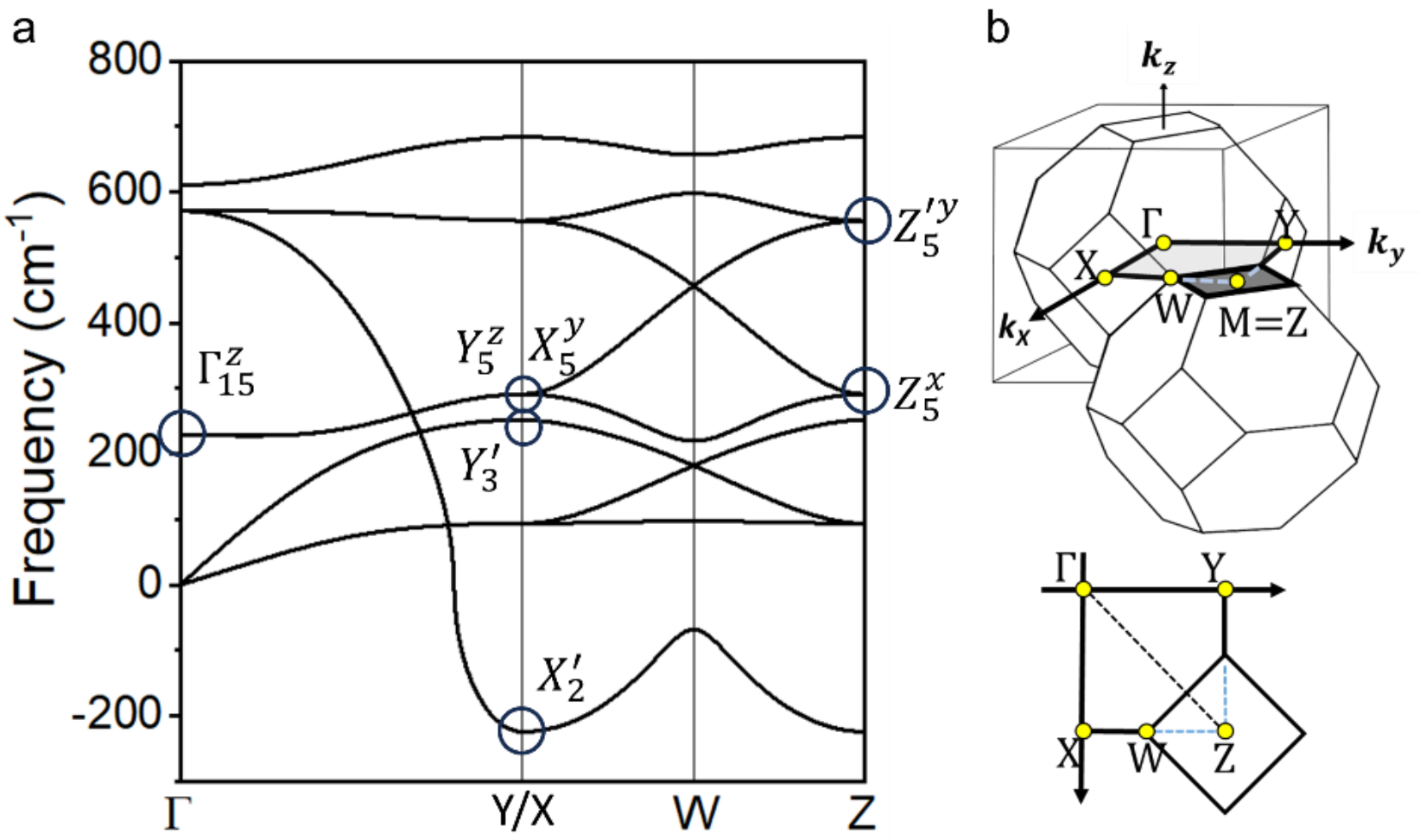

Figure S6. (a) Phonon dispersion of the primitive cubic phase of HfO_2, with the frequencies of eight modes driving the transformation to the ferroelectric $Pca2_1$ phase highlighted by circles. (b) Brillouin zone of the face-centered cubic (FCC) crystal structure.

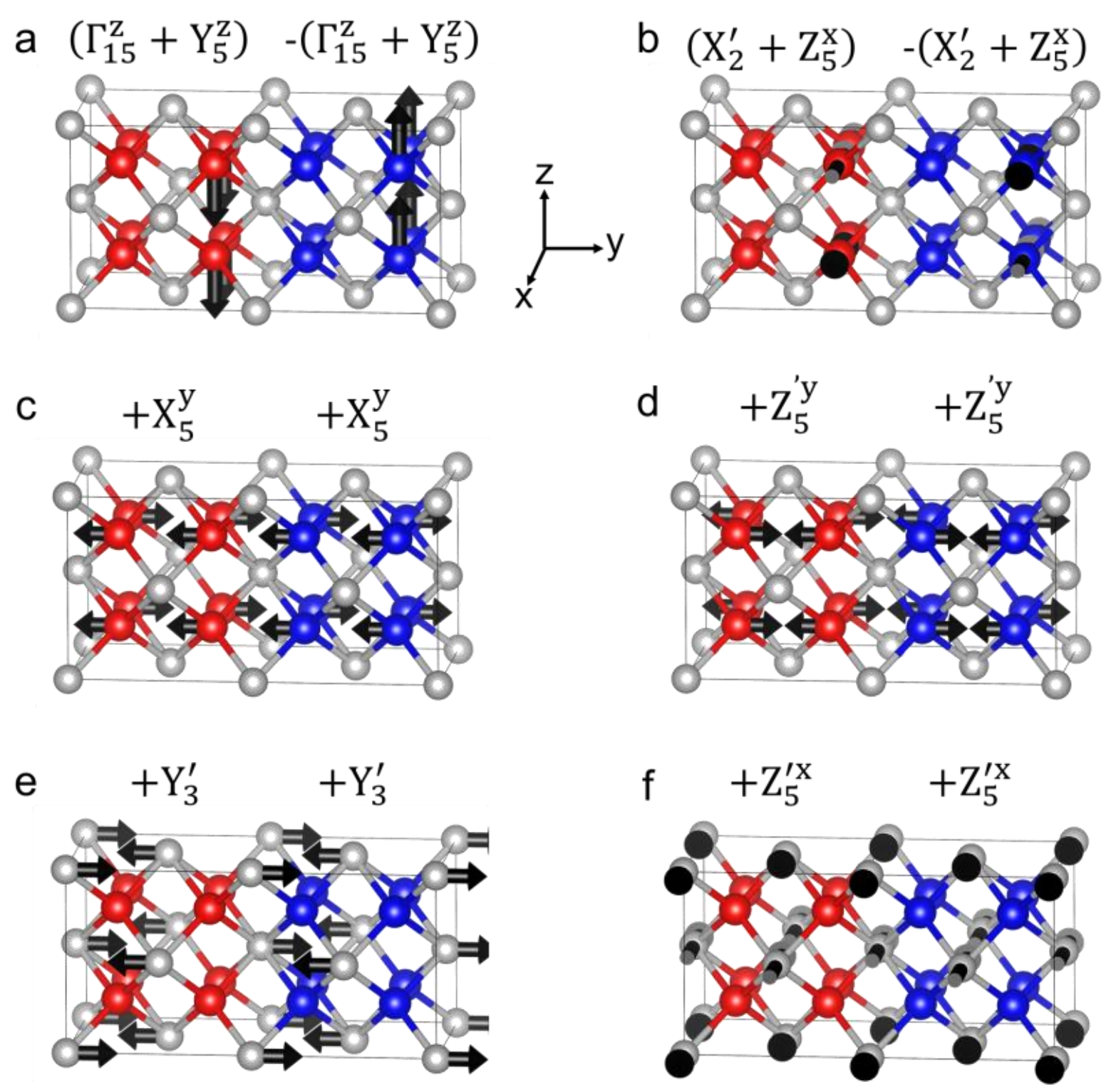

Figure S7. Modes transforming the cubic phase into the HE-$Pbca$ Hf-center phase at [010] U/D_{xy} DWs. Atomic displacements corresponding to reducible mixed polar (Γ_{15}^{z}) and antipolar (Y_5^{z}) modes (a) and unstable (X_2') and stable (Z_5^{x}) antipolar modes (b), which reverse sign during DW formation, in the cell-doubled conventional cubic unit cell along [010] direction, whereas irreducible modes that do not change sign are shown in (c–f).

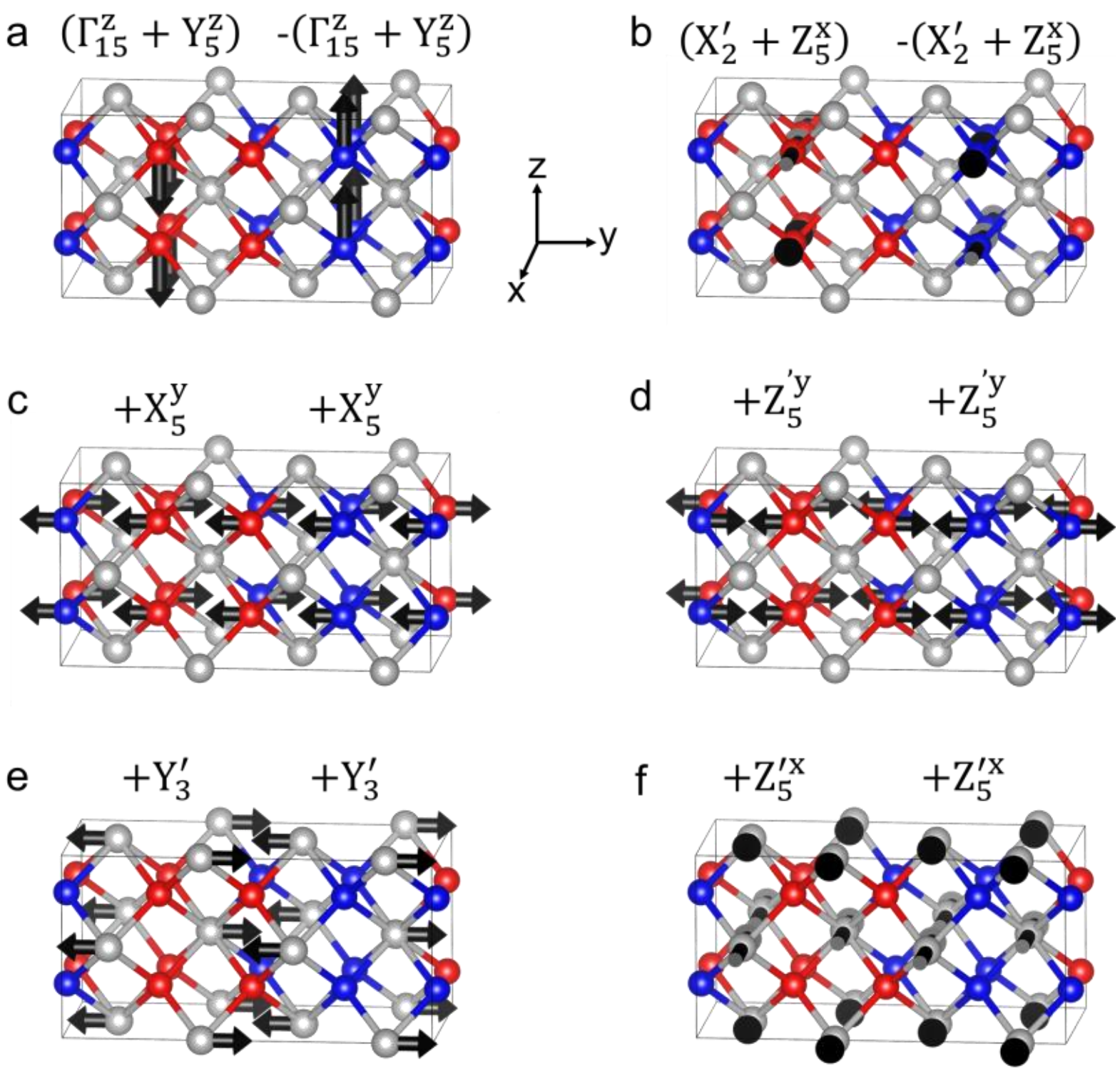

Figure S8. Modes transforming the cubic phase into the HE-$Pbca$ SL-center phase at [010] U/D_{xy} DWs. Atomic displacements corresponding to reducible mixed polar (Γ_{15}^{z}) and antipolar (Y_5^{z}) modes (a) and unstable (X_2') and stable (Z_5^{x}) antipolar modes (b), which reverse sign during DW formation, in the cell-doubled conventional cubic unit cell along [010] direction, whereas irreducible modes that do not change sign are shown in (c–f).

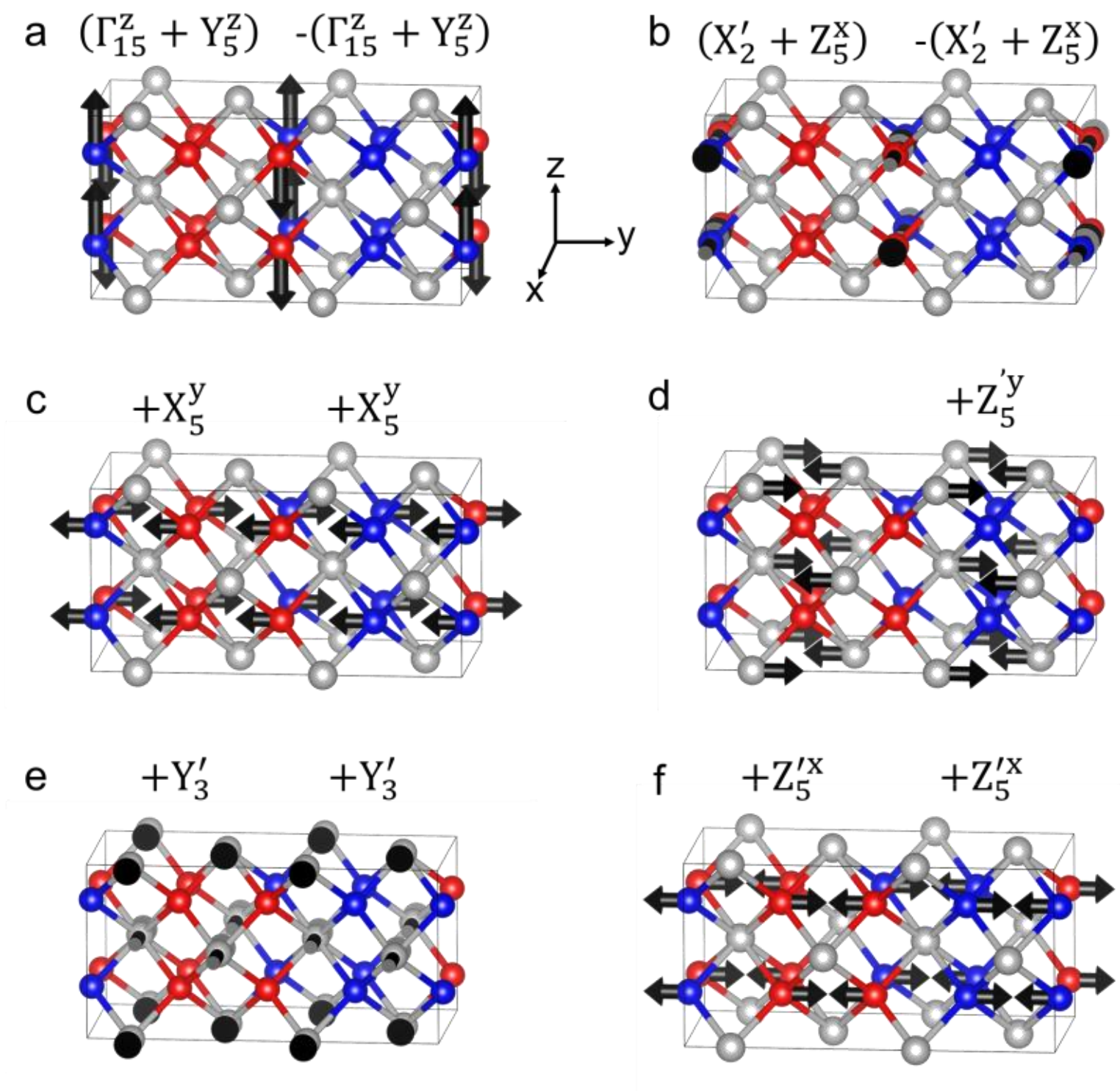

Figure S9. Modes transforming the cubic phase into the LE-$Pbca$ FL-centered phase at [010] U/D_{xy} DWs. Atomic displacements corresponding to reducible mixed polar (Γ_{15}^{z}) and antipolar (Y_5^z) modes (a) and unstable (X_2') and stable (Z_5^x) antipolar modes (b), which reverse sign during DW formation, in the cell-doubled conventional cubic unit cell along [010] direction, whereas irreducible modes that do not change sign are shown in (c–f).

Estimation of gradient energy

The gradient energy (E_{grad}) can be expressed in terms of the curvatures of the phonon bands for each mode along the wavevector in the domain-wall direction[2],

$E_{grad} = \sum_{\nu} \mu_{\nu} \omega_{0\nu} g_{\nu} \sum_{q} Q_{\nu}^{2} q^{2}$, (S3)

where, μ_ν, $\omega_{0\nu}$, and g_ν denote the effective ionic mass, absolute frequency, and the phonon band gradient of mode ν, respectively. Q_ν is the amplitude (listed in **Figure S5b**) of mode ν in reciprocal space, and q denotes the wavevector along the DW direction. In our case we estimated q equivalent to the super-cell used in our calculations, $\omega_{0\nu}$ from phonon dispersion (**Figure S6a**).

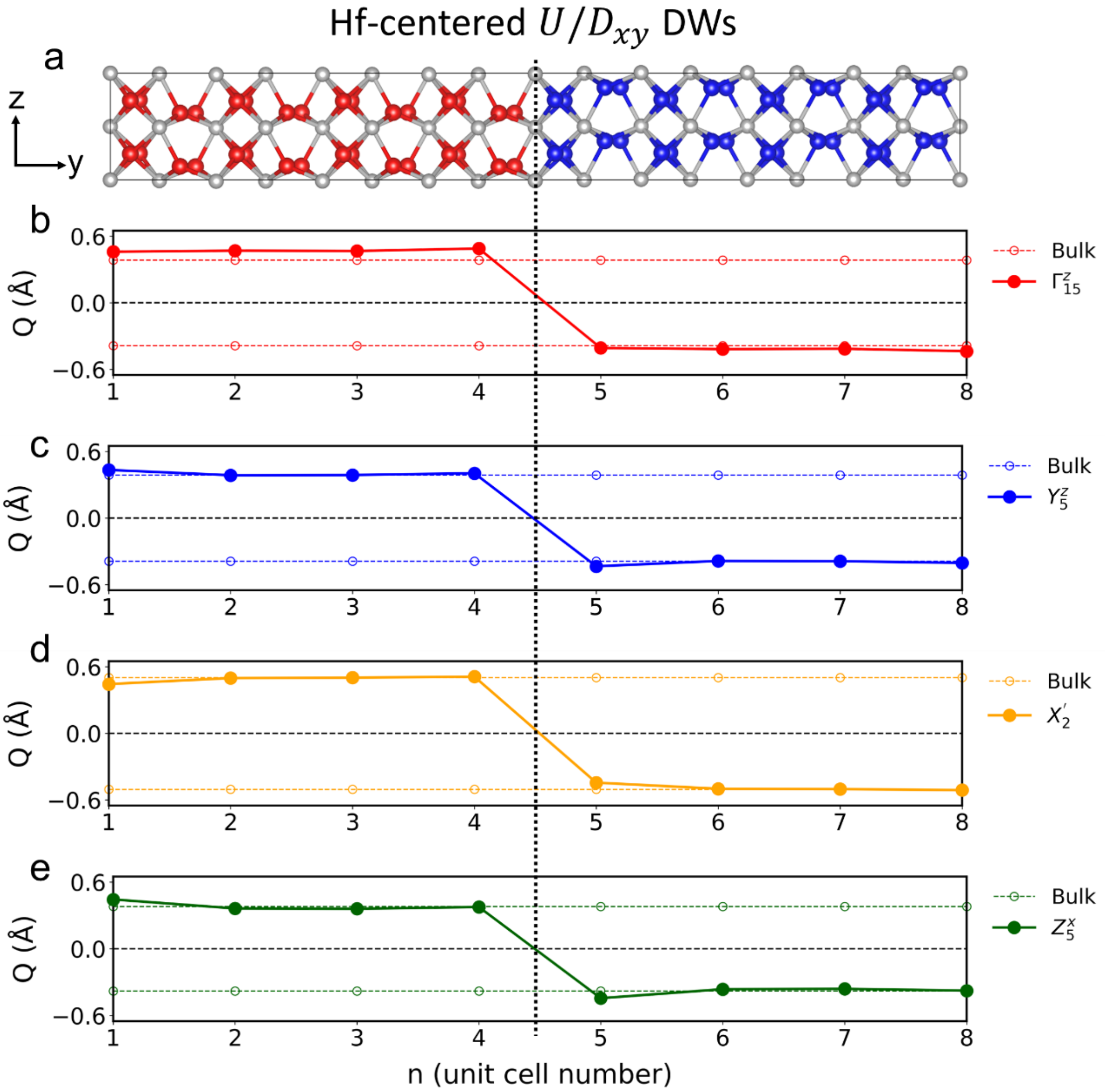

Figure S10. Atomic structures (a) and corresponding mode amplitudes of Γ_{15}^{z} (b), Y_5^z (c), X_2' (d), and Z_5^x (e) in Hf-centered U/D_{xy} DWs within a 1×8×1 supercell along the [010] direction. Solid and dashed lines denote amplitudes of modes in the relaxed and unrelaxed configurations, respectively.

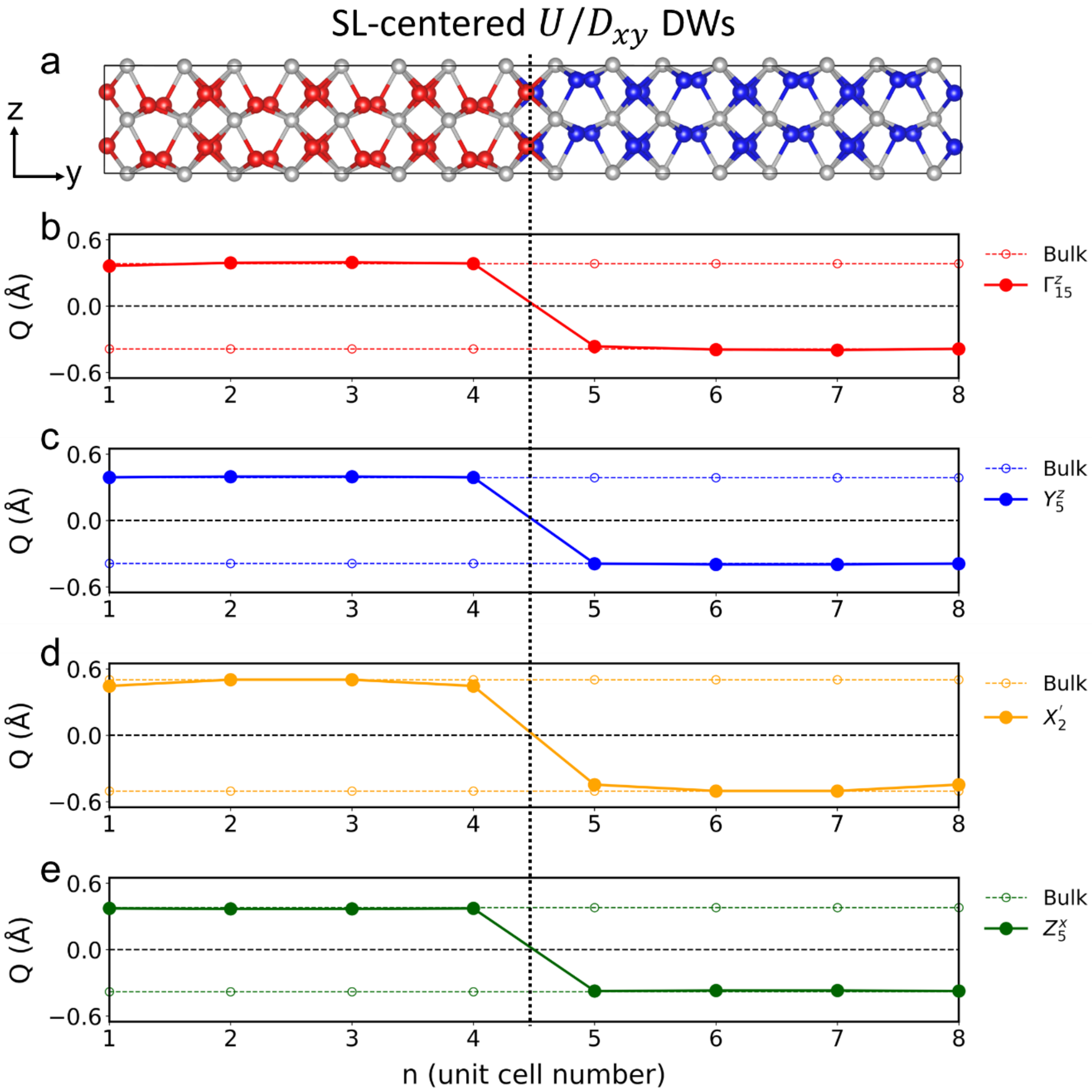

Figure S11. Atomic structures (a) and corresponding mode amplitudes of Γ_{15}^{z} (b), Y_5^z (c), X_2' (d), and Z_5^x (e) in SL-centered U/D_{xy} DWs within a 1×8×1 supercell along the [010] direction. Solid and dashed lines denote amplitudes of modes in the relaxed and unrelaxed configurations, respectively.

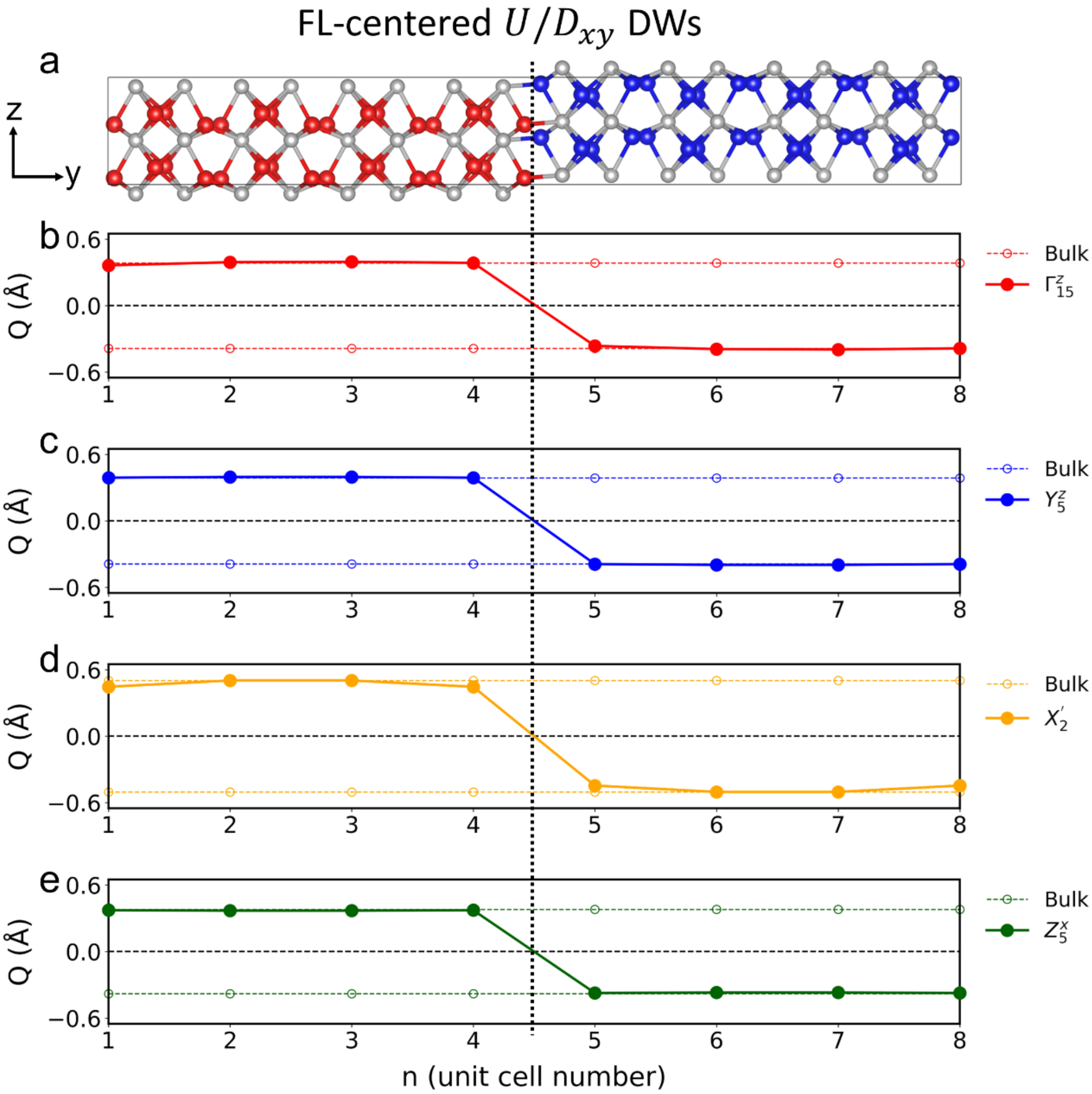

Figure S12. Atomic structures (a) and corresponding mode amplitudes of Γ_{15}^z (b), Y_5^z (c), X_2' (d), and Z_5^x (e) in FL-centered U/D_{xy} DWs within a 1×8×1 supercell along the [010] direction. Solid and dashed lines denote amplitudes of modes in the relaxed and unrelaxed configurations, respectively.

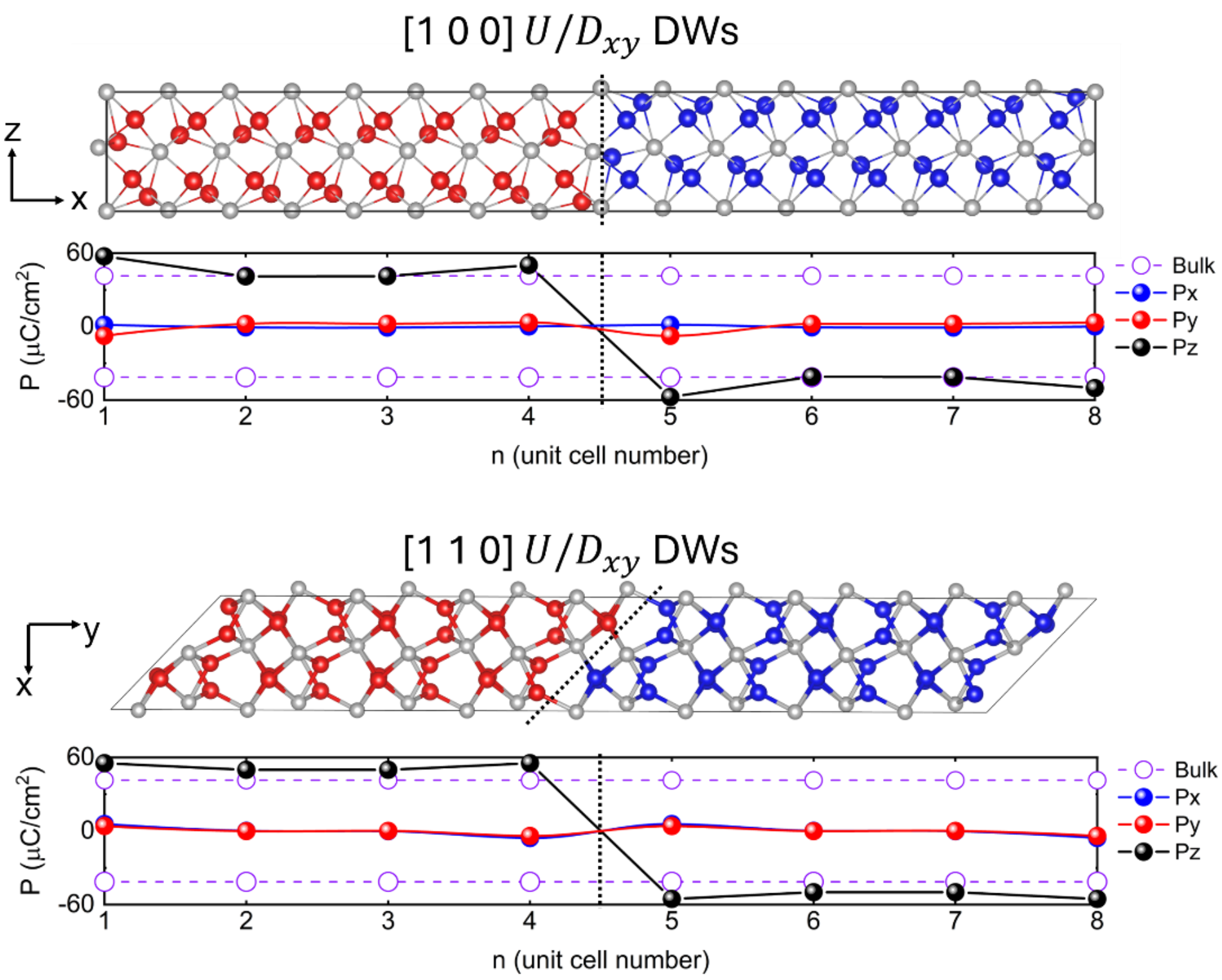

Figure S13. Atomic structures and the corresponding polarization in each unit cell of a 1×8×1 supercell for [1 0 0] direction (a, b) and [1 1 0] direction (c, d) U/D_{xy} domain walls. Solid black lines show the polarization in each unit cell for the relaxed domain wall configuration, while purple dashed lines indicate the bulk polarization.

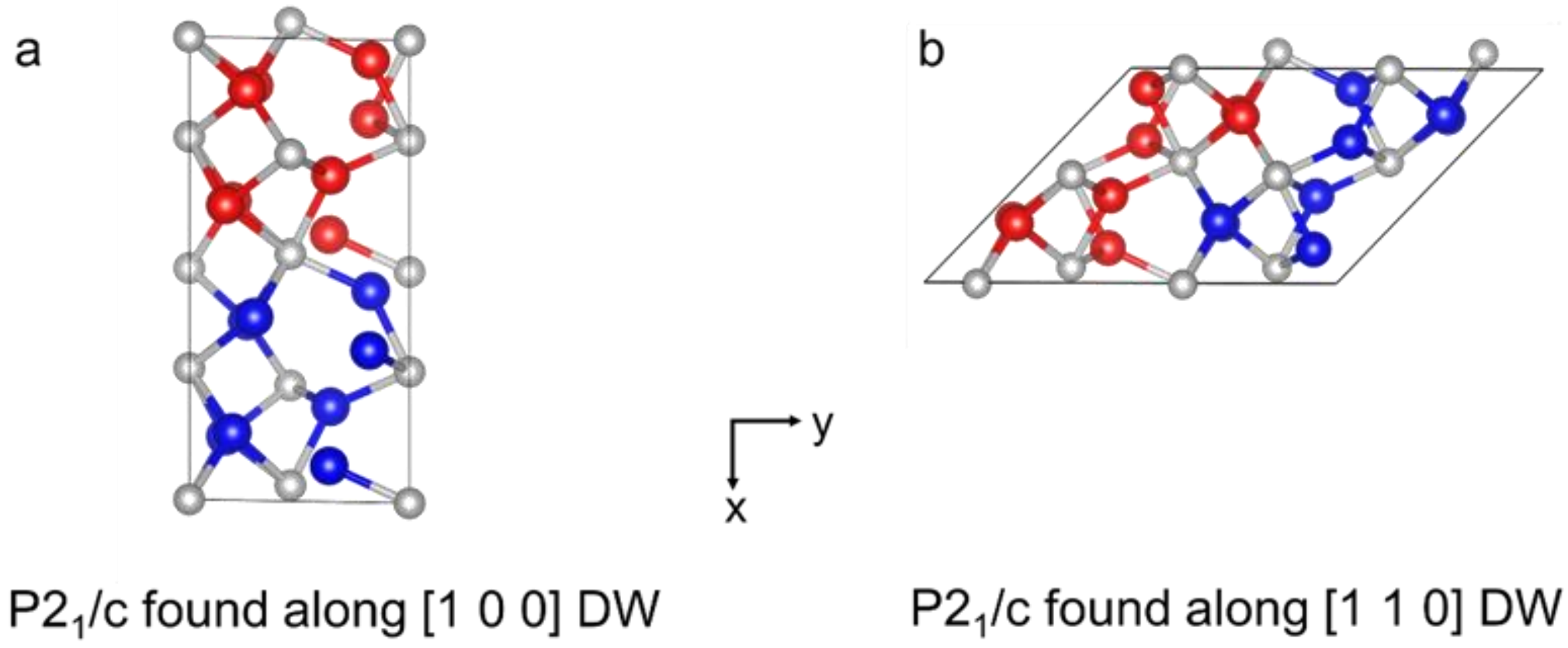

Figure S14. Atomic structures of the 24-atom unit cell of the $P2_1/c$ phase, stabilized at [100] (a) and [110] (b) DWs. Although both structures belong to the same space group, they exhibit distinct atomic positions and lattice parameters.

Table S2. Lattice parameters and polarization of different phases stabilized in bulk HfO_2, along with their energies relative to the ground-state monoclinic $P2_1/c$ phase.

Structure	Characteristic	a(Å)	b(Å)	c(Å)	Monoclinic angle (α°)	$E - E_{GS}$ (meV/f.u.)	Polarization ($\mu C/cm^2$)
$P2_1/c$	Ground state	5.198	5.147	5.329	99.7	0.00	0
$P2_1/c$	[1 0 0] DW	10.506	5.015	5.098	91.0	207	0
$P2_1/c$	[1 1 0] DW	7.303	10.042	5.116	133.8	207	0
Pbca	[0 1 0] DW	5.273	10.112	5.085	-	72	0
$Pca2_1$	Ferroelectric	5.273	5.056	5.085	-	83	41.5
$Fm\bar{3}m$	Reference phase	5.079	5.079	5.079	-	261	0

SI Note-1: The poscar files of P21/c phases at [1 0 0] and [1 1 0] DWs

POSCAR of P21/c phsae at [1 0 0] DWs

```
1.0
    10.5064544678    0.0000000000    0.0000000000
     0.0886700024    5.0140141863    0.0000000000
     0.0000244404   -0.0000343826    5.0984568594
  Hf  O
   8  16
Direct
   0.000000000   0.000000000   0.000000000
   0.249937996   0.458781987   0.998160005
   0.215930000   0.000381000   0.498162001
   0.465882987   0.459163010   0.500002980
   0.500003994   0.999998987   0.026533000
   0.749954998   0.458779007   0.028374000
   0.715947986   0.000379000   0.528376997
   0.965883970   0.459160000   0.526535988
   0.294411987   0.641323984   0.607702017
   0.112306997   0.262910992   0.762696981
   0.171480998   0.817826986   0.107703000
   0.353585005   0.196251005   0.262697011
   0.043430999   0.824036002   0.571989000
   0.362313002   0.164104000   0.763025999
   0.422459990   0.635132015   0.071992002
   0.103577003   0.295058012   0.263024002
   0.922454000   0.635124981   0.954550028
   0.603570998   0.295056999   0.763509989
   0.543424010   0.824029028   0.454546988
   0.862308025   0.164102003   0.263511986
   0.671473026   0.817837000   0.918835998
   0.853577971   0.196250007   0.763839006
   0.794403970   0.641332984   0.418835014
   0.612299979   0.262910992   0.263839006
```

POSCAR of P21/c phsae at [1 1 0] DWs

```
1.00000000000000
  7.3051090240000001   0.0000000000000000   0.0000000000000000
 -6.9983041477999999   7.2986620583999997   0.0000000000000000
  0.0000000000000000   0.0000000000000000   5.0853381156999999
Hf   O
  8   16
Direct
  0.0060870000000008  0.1304219960000026  0.9634079929999970
  0.4985269900000020  0.1053069980000032  0.9874759909999966
  0.4351269900000005  0.3447870019999968  0.4874840080000027
  0.9275559780000009  0.3196739850000014  0.4634140129999977
  0.0061019999999985  0.6304360029999998  0.0366539990000021
  0.4985420109999978  0.6053220029999977  0.0125829999999993
  0.4351210000000023  0.8447930219999975  0.5125910040000008
  0.9275709989999967  0.8196769950000018  0.5366600159999990
  0.5936009879999986  0.2475669980000035  0.5881389979999980
  0.2248030009999979  0.3816649910000010  0.7334679960000017
  0.3400569860000004  0.2025430050000026  0.0881419999999977
  0.7088590259999989  0.0684410040000003  0.2334679960000017
  0.0943230020000030  0.0856029990000025  0.5684120060000026
  0.7248600129999971  0.0845469980000004  0.7344530219999967
  0.8393409849999998  0.3645060059999992  0.0684159990000026
  0.2088039960000003  0.3655610080000002  0.2344519939999969
  0.8393340109999983  0.8645039800000021  0.9316530230000026
  0.2088019999999986  0.8655599949999981  0.7656160000000014
  0.0943190010000023  0.5855960250000010  0.4316479860000015
  0.7248510120000020  0.5845419759999970  0.2656170130000035
  0.3400599960000008  0.7025380130000016  0.9119240050000030
  0.7088490129999983  0.5684360269999971  0.7665979860000007
  0.5935980080000007  0.7475609780000028  0.4119209950000027
  0.2248049969999997  0.8816660049999996  0.2665989999999994
```

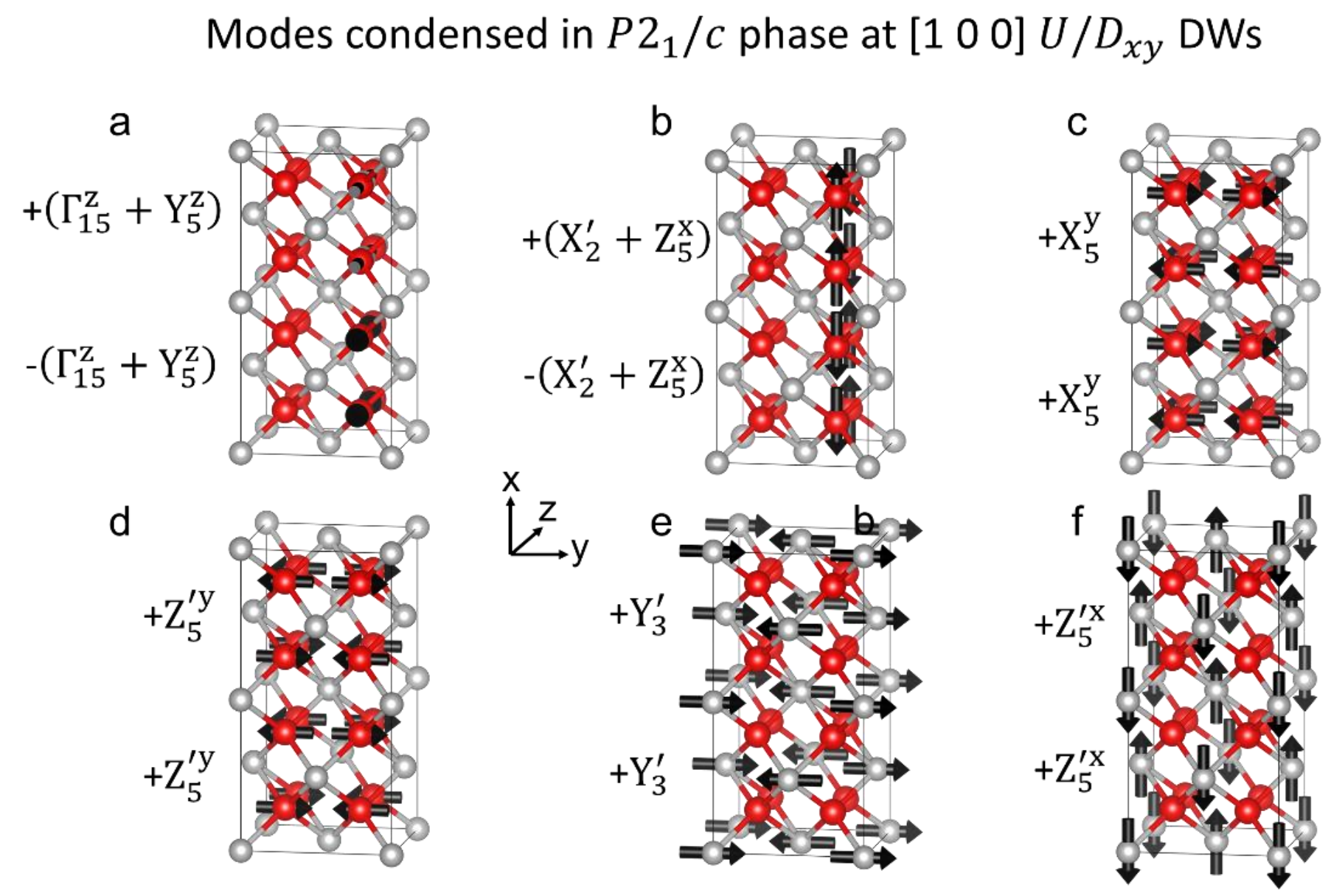

Figure S15. Modes transforming the cubic phase into the $P2_1/c$ phase at [100] U/D_{xy} DWs. Atomic displacements corresponding to reducible mixed polar (Γ_{15}^{z}) and antipolar (Y_5^{z}) modes (a) and unstable (X_2') and stable (Z_5^{x}) antipolar modes (b), which reverse sign during DW formation, in the cell-doubled conventional cubic unit cell along [100] direction, whereas irreducible modes that do not change sign are shown in (c–f).

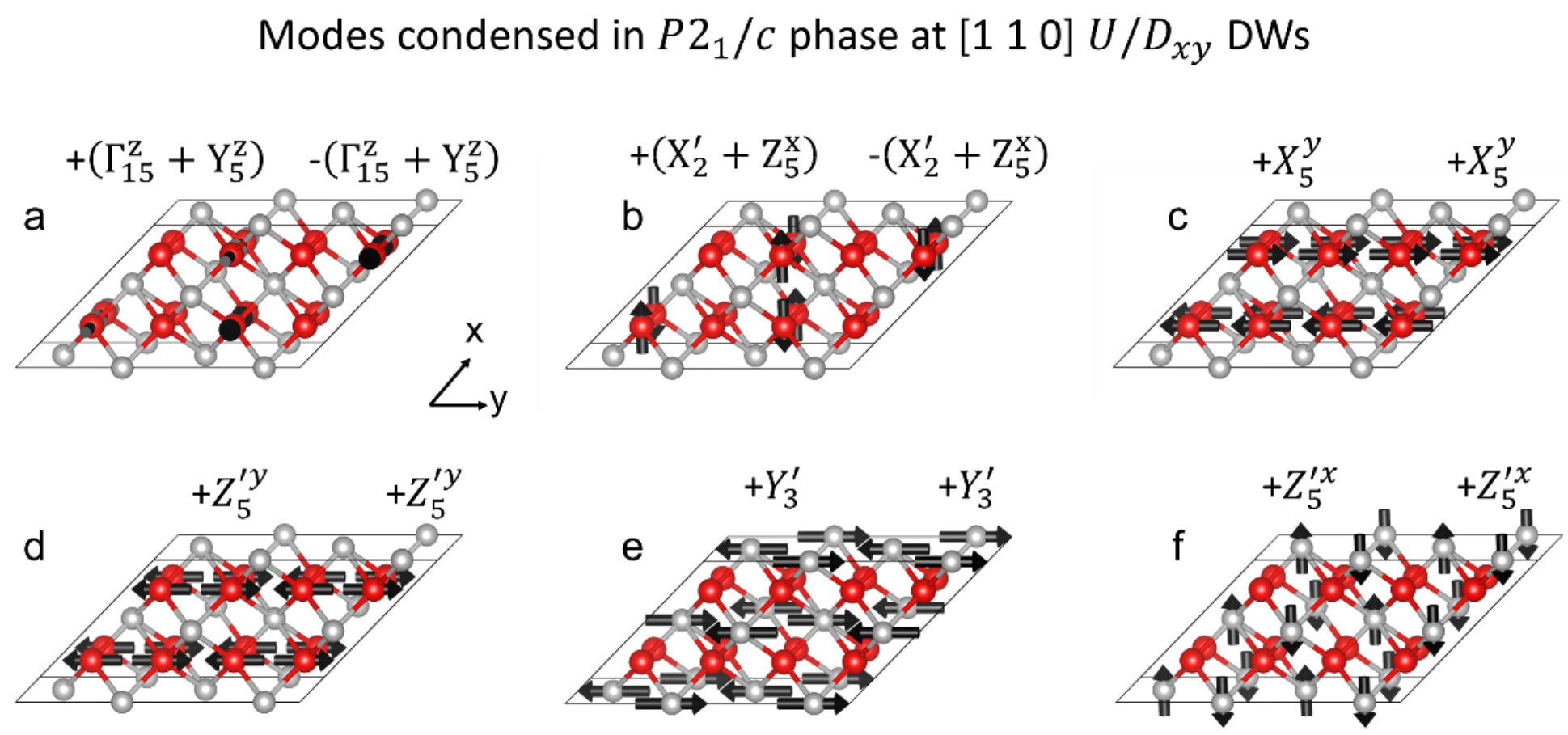

Figure S16. Modes transforming the cubic phase into the $P2_1/c$ phase at [110] U/D_{xy} DWs. Atomic displacements corresponding to reducible mixed polar (Γ_{15}^{z}) and antipolar (Y_5^{z}) modes (a) and unstable (X_2') and stable (Z_5^{x}) antipolar modes (b), which reverse sign during DW formation, in the diagonally tilted cell-doubled conventional cubic unit cell along [110] direction, whereas irreducible modes that do not change sign are shown in (c–f).

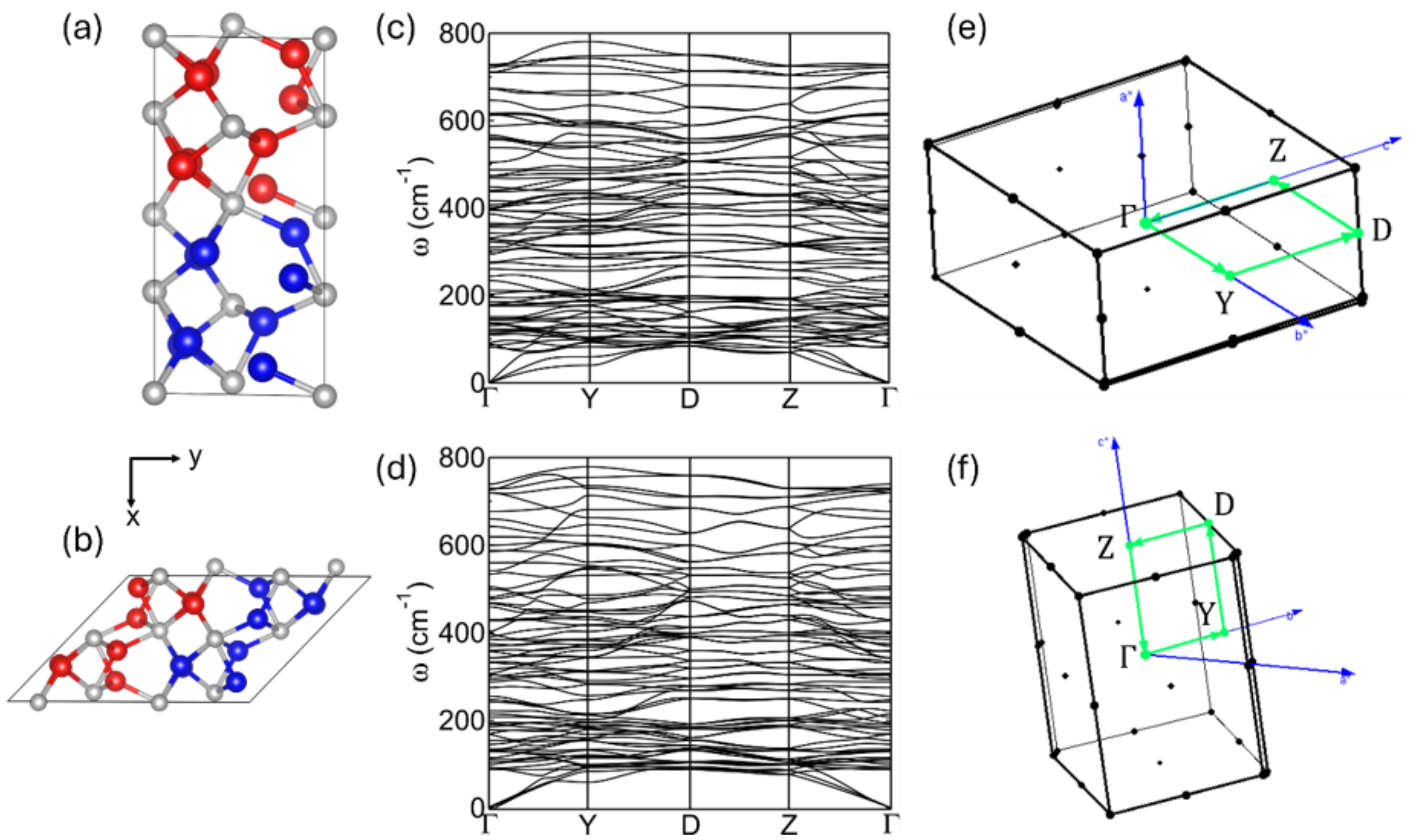

Figure S17. Atomic structures of the $P2_1/c$ phase at the [1 0 0] (a) and [1 1 0] (b) domain walls (DWs). The phonon dispersions of the $P2_1/c$ phase for the [1 0 0] (c) and [1 1 0] (d) DWs exhibit only positive frequencies, confirming their dynamical stability. The corresponding Brillouin zones are depicted in (e) and (f), highlighting the high-symmetry paths with green arrows used for the phonon dispersion calculations.

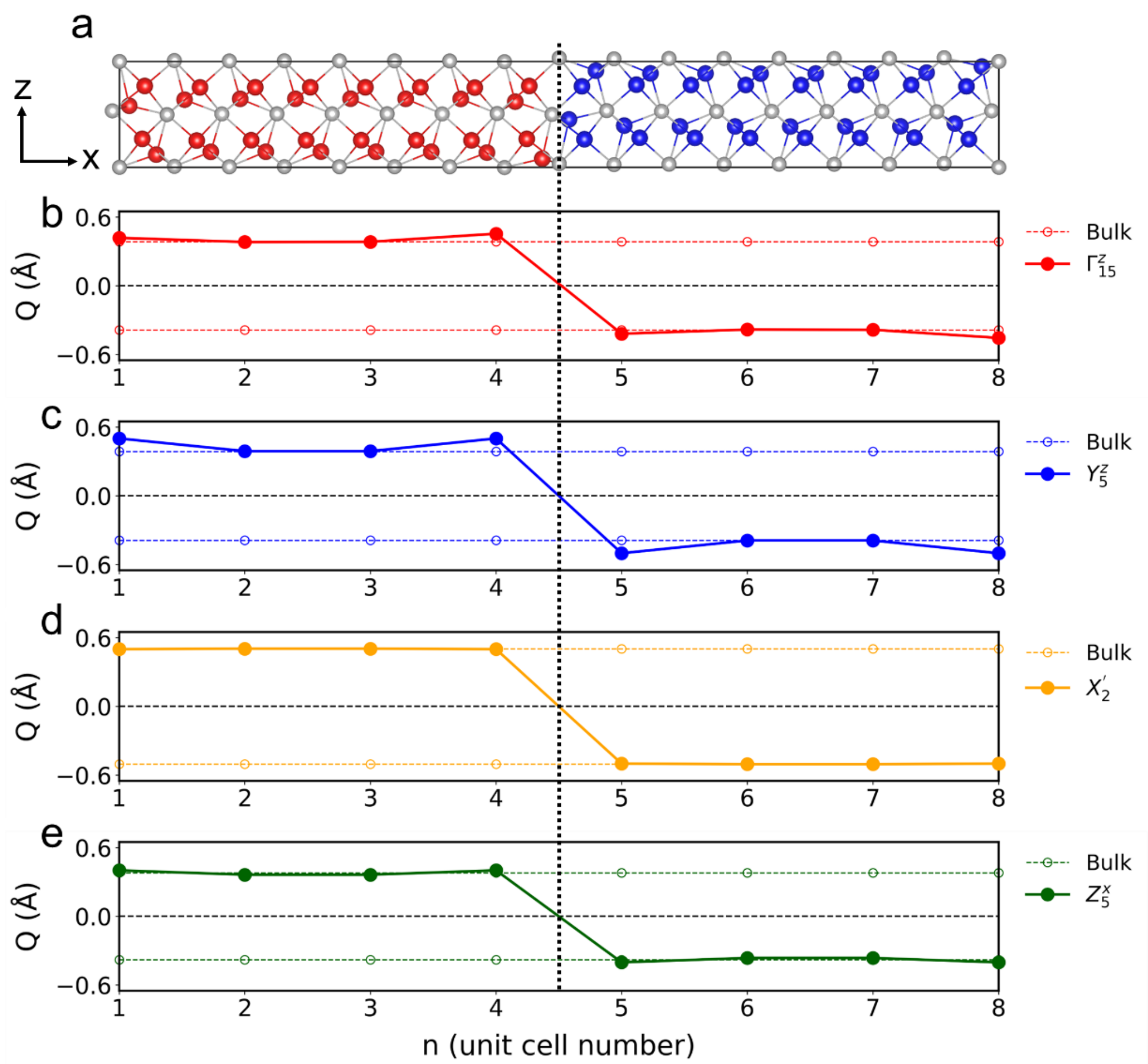

Figure S18. Atomic structures (a) and corresponding mode amplitudes of Γ_{15}^{z} (b), Y_5^z (c), X_2' (d), and Z_5^x (e) in the U/D_{xy} DWs within a 8×1×1 supercell along the [1 0 0] direction. Solid and dashed lines denote amplitudes of modes in the relaxed and unrelaxed configurations, respectively.

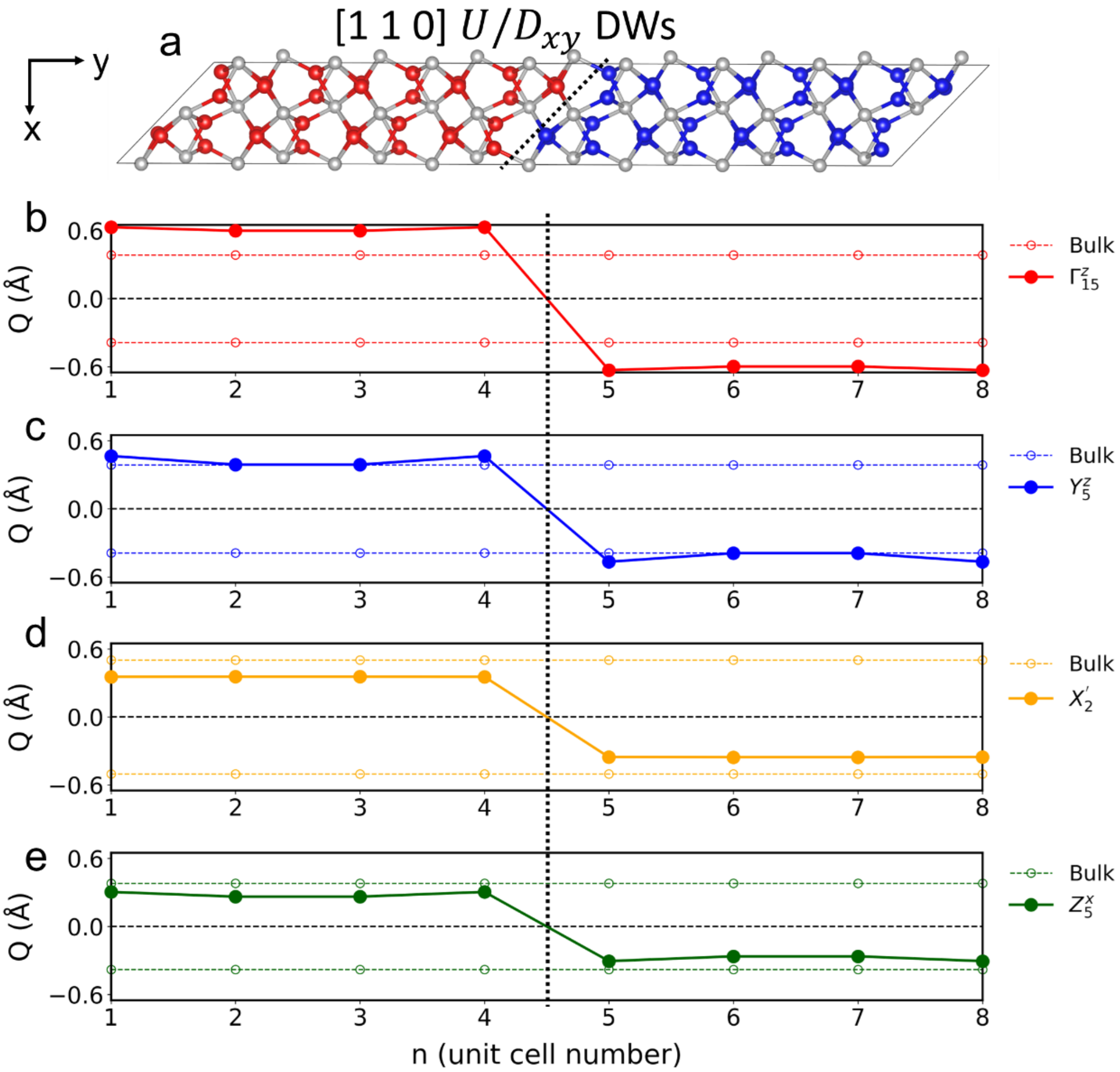

Figure S19. Atomic structures (a) and corresponding mode amplitudes of Γ_{15}^{z} (b), Y_5^z (c), X_2' (d), and Z_5^x (e) in the U/D_{xy} DWs within a 1×8×1 supercell along the [1 1 0] direction. Solid and dashed lines denote amplitudes of modes in the relaxed and unrelaxed configurations, respectively.

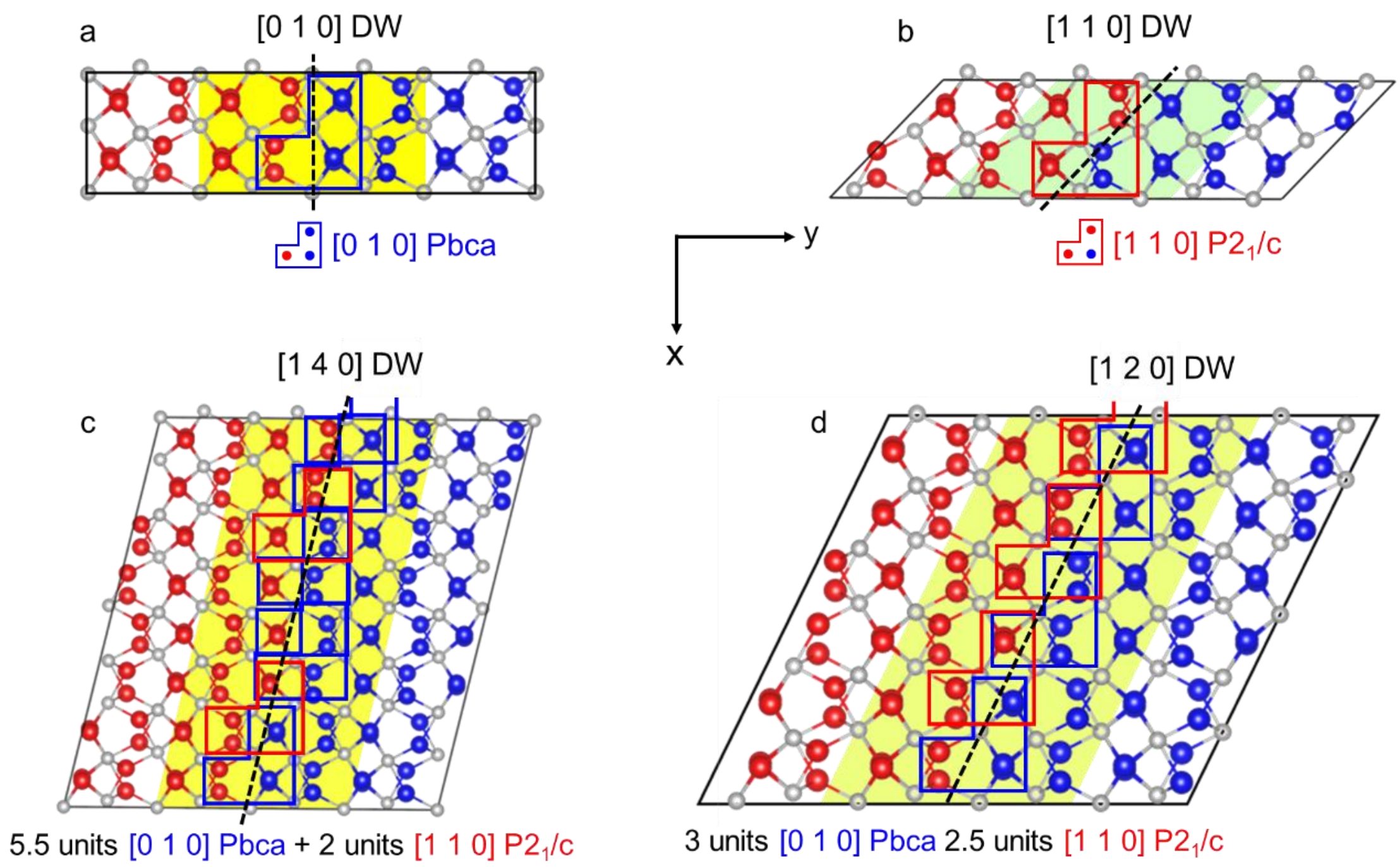

Figure S20. Atomic structures of DWs [0 1 0] (a), [1 1 0] (b), [1 4 0] (c) and [1 2 0] (d). The shaded regions with different colors at DWs depict the crystallized DW structures. The crystal structure of the [0 1 0] DW is a perfect $Pbca$ (a) and of the [1 1 0] DW is perfect $P2_1/c$ (b). The smallest structural units used to count the [0 1 0] $Pbca$ and [1 1 0] $P2_1/c$ phases at the DWs are indicated by solid blue and solid red L-shaped regions, respectively. The DW structures of [1 4 0] and [1 2 0] DWs, contain components of the [0 1 0] $Pbca$ and [1 1 0] $P2_1/c$ phases in proportions determined by their directional ratios.

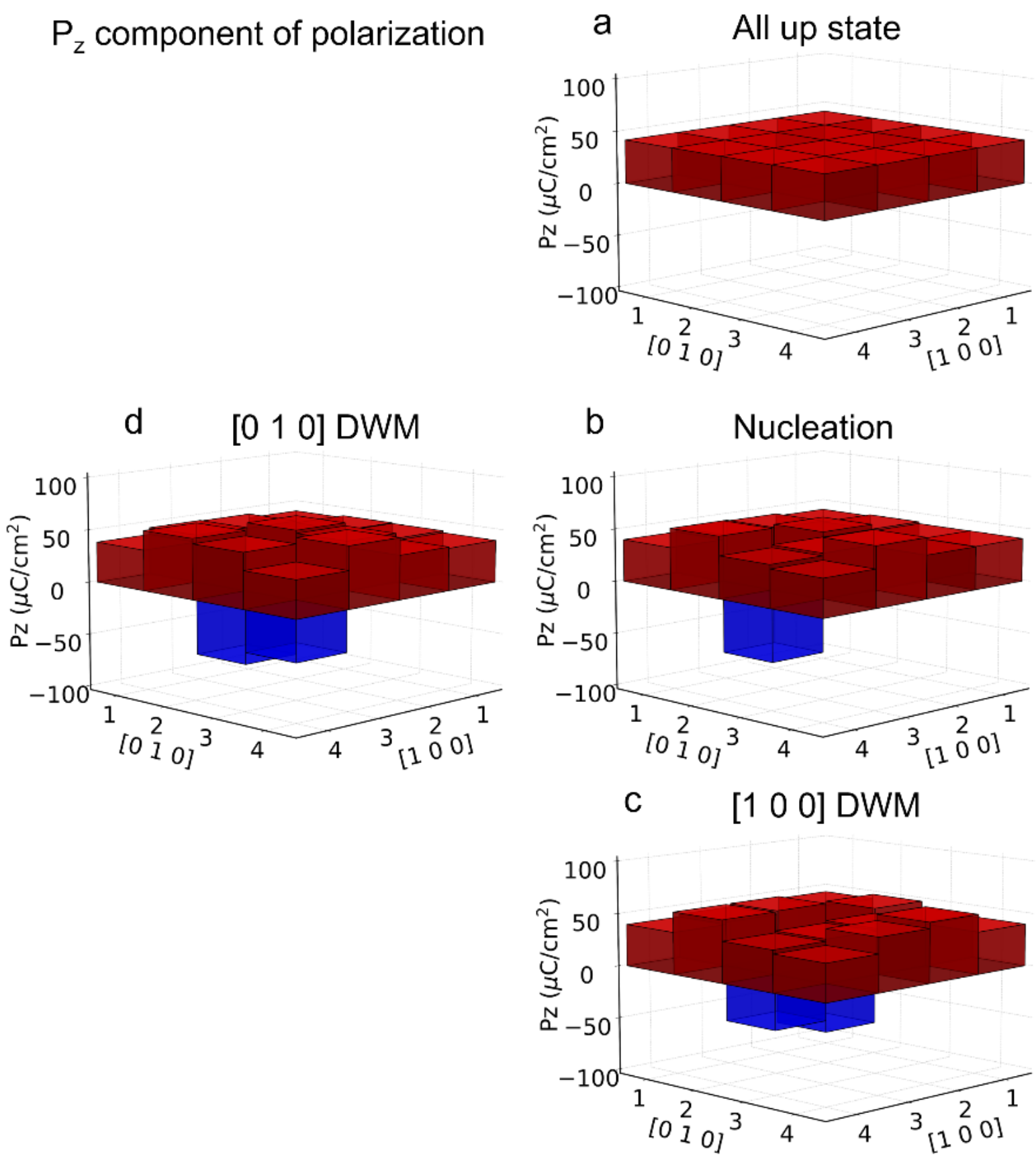

Figure S21. Polarization profile in each unit cell of all up-polarized state (a), nucleation state with one down-polarized unit cell (b), and adjacent down-polarized unit cell along [1 0 0] (c), [0 1 0] (d), and [1 1 0] (e) directions in 4x4x1 supercell. Red and blue bars depict the up (+P_z) and down (-P_z) polarization in their respective unit cells.

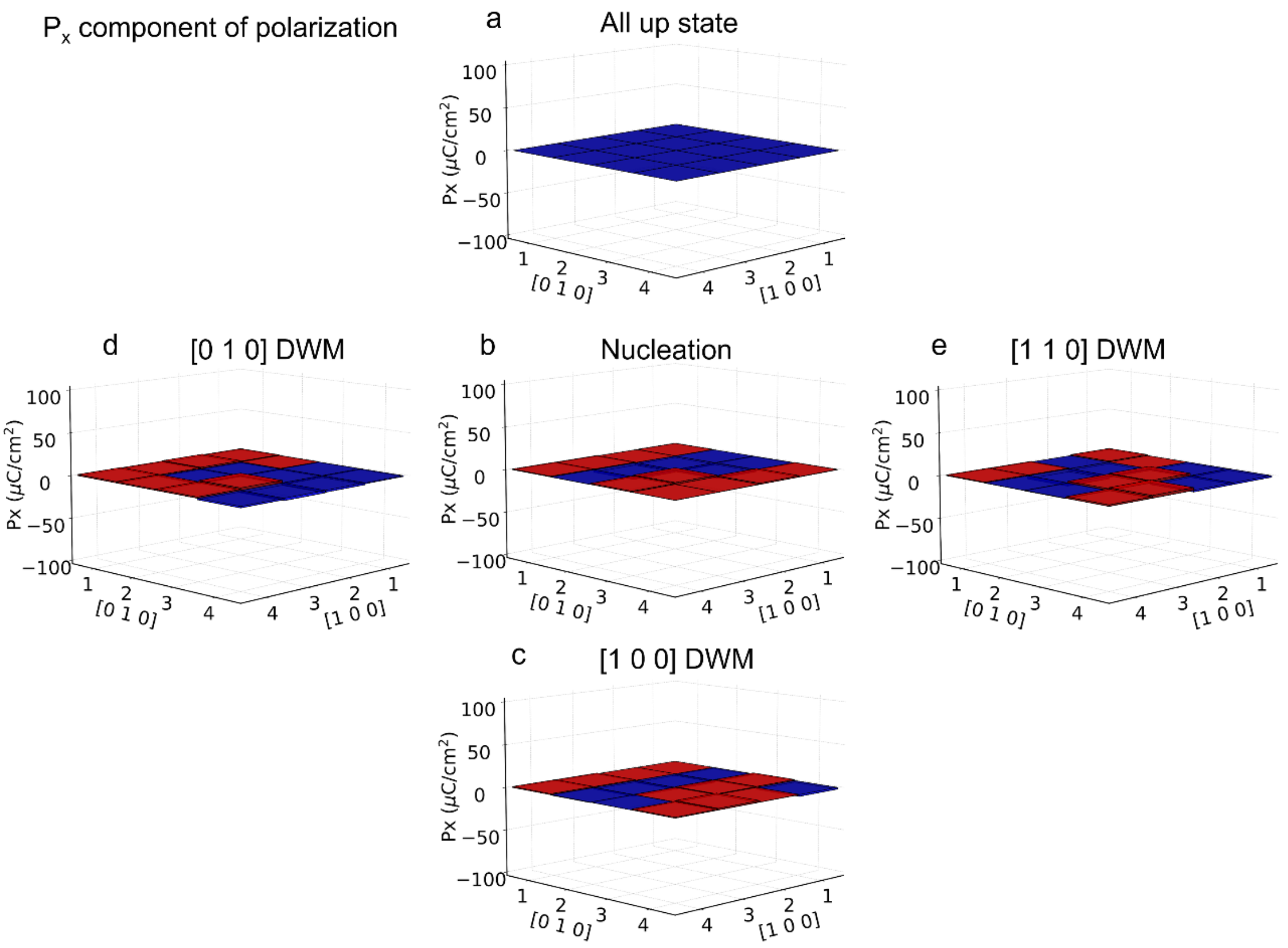

Figure S22. Nagligibly zero P_x component of polarization in each unit cell of all up-polarized state (a), nucleation state with one down-polarized unit cell (b), and adjacent down-polarized unit cell along [1 0 0] (c), [0 1 0] (d), and [1 1 0] (e) directions in 4x4x1 supercell. Red and blue bars depict the $+P_x$ and $-P_x$ polarization in their respective unit cells.

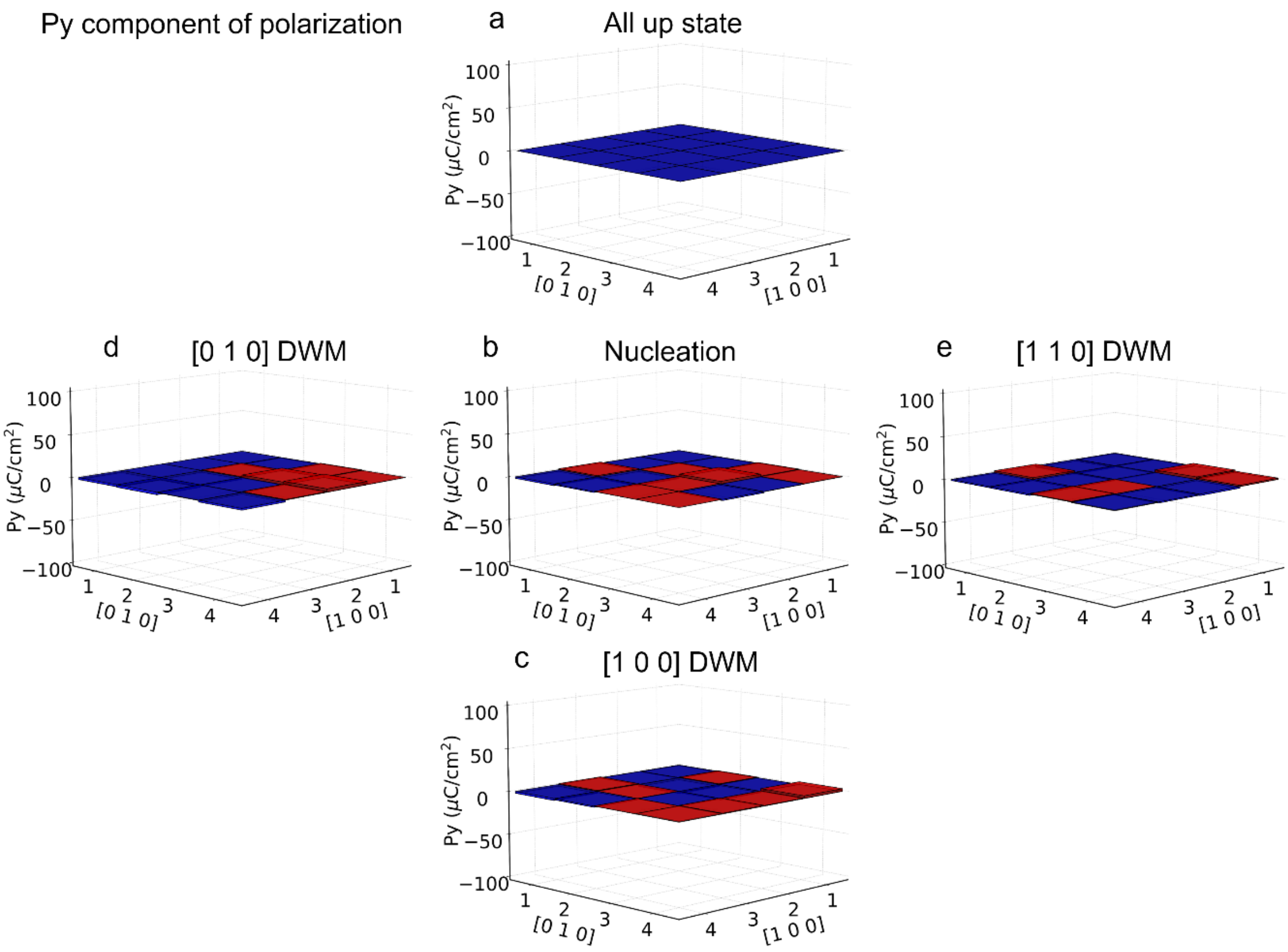

Figure S23. Nagligibly zero P_y component of polarization in each unit cell of all up-polarized state (a), nucleation state with one down-polarized unit cell (b), and adjacent down-polarized unit cell along [1 0 0] (c), [0 1 0] (d), and [1 1 0] (e) directions in 4x4x1 supercell. Red and blue bars depict the $+P_y$ and $-P_y$ polarization in their respective unit cells.

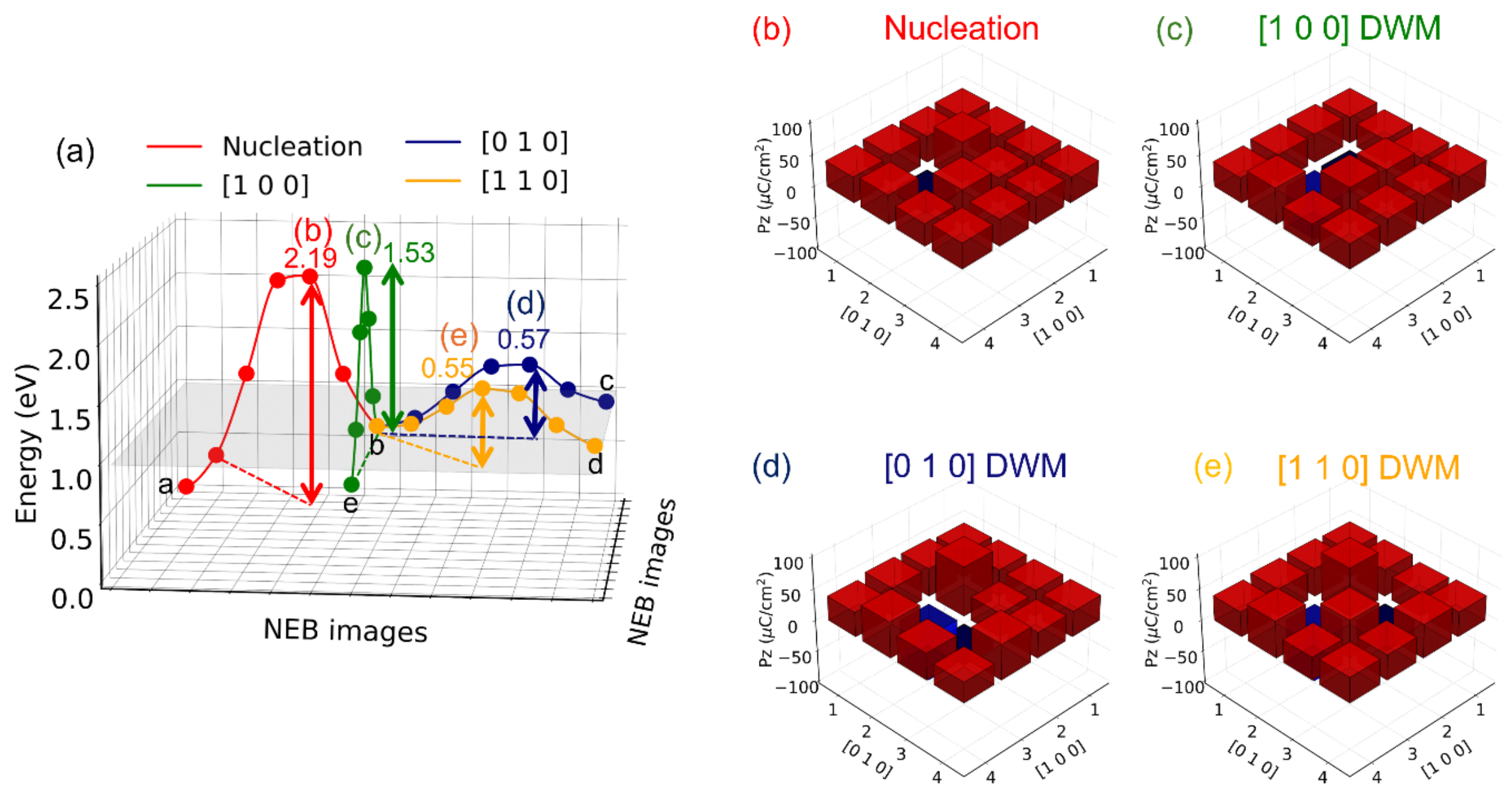

Figure S24. Estimated energy barriers separating distinct domain wall configurations (a). The polarization profiles at the transition state for domain nucleation (b), [1 0 0] DW motion (c), [0 1 0] DW motion (d), and [1 1 0] DW motion (e). The red and blue bars represent the polarization of up- and down-polarized unit cells, respectively. At the transition state, only the unit cell undergoing polarization switching exhibits suppressed polarization, whereas the neighboring unit cells retain substantial polarization. This indicates that the stability of the neighboring unit cells is largely preserved during the switching process.

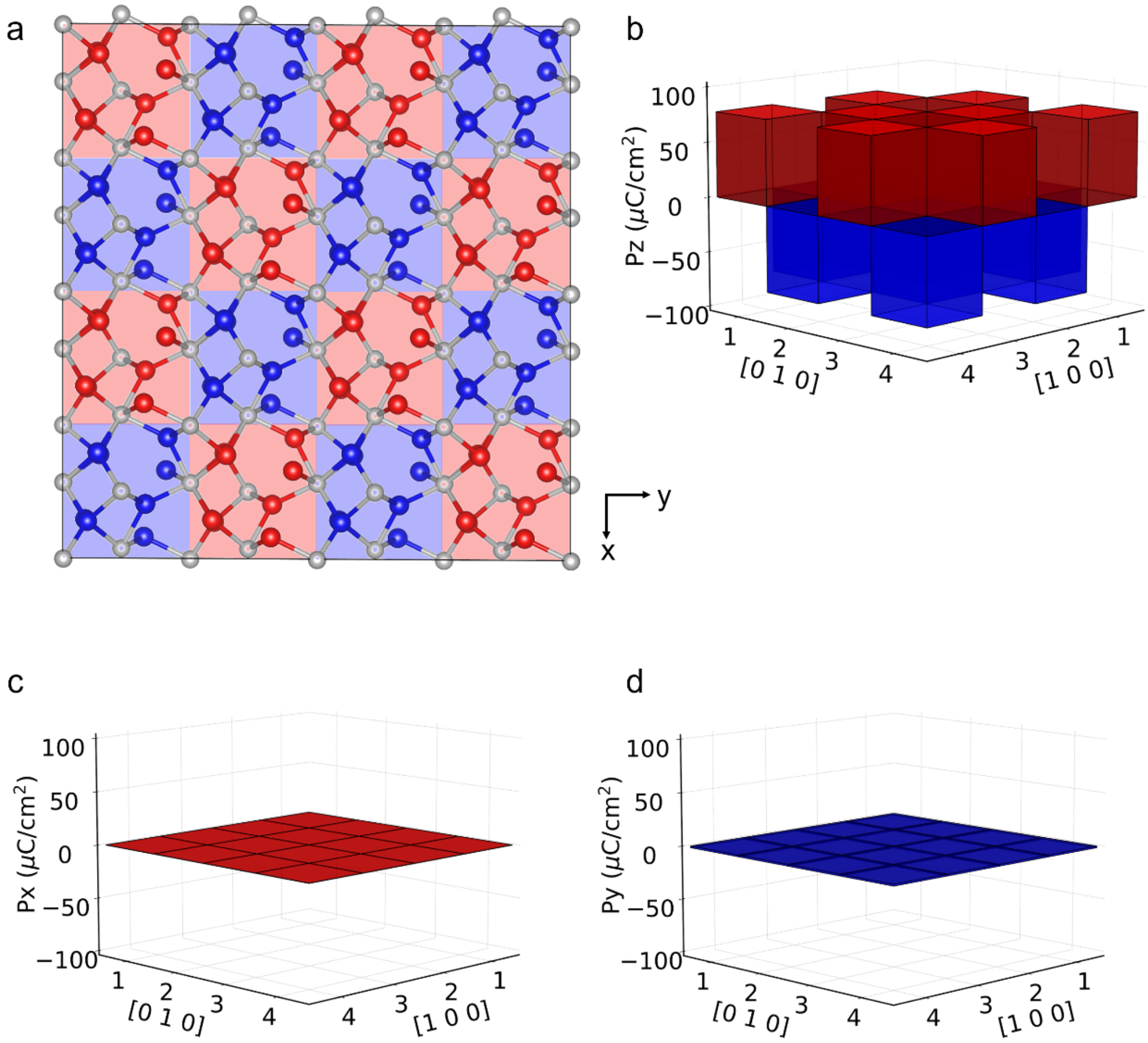

Figure S25. (a) Atomic structure of a 4×4×1 supercell with alternating U (red) and D_{xy} (blue) unit cells in the xy plane, forming a chessboard-type arrangement of up (+P_z) and down (-P_z) polarized domains. (b) Polarization profile in each unit cell, the absolute value of P_z component of polarization does not suppress across the unit cell and remains almost identical. While the P_x (c) and P_y (d) components are nearly zero.

References

[1] X. Wang, T. Xu, F. Xuan, C. Chen, T. Shimada, T. Kitamura, *J. Appl. Phys.* **2019**, *126*, 174107.

[2] P. Kumar, D. Gupta, J. H. Lee, *Phys. Rev. Lett.* **2025**, *134*, 166101.
